# Comparative genomic analysis of *Brevibacterium* strains: insights into key genetic determinants involved in adaptation to the cheese habitat

**DOI:** 10.1186/s12864-017-4322-1

**Published:** 2017-12-07

**Authors:** Nguyen-Phuong Pham, Séverine Layec, Eric Dugat-Bony, Marie Vidal, Françoise Irlinger, Christophe Monnet

**Affiliations:** 1 UMR GMPA, AgroParisTech, INRA, Université Paris-Saclay, 78850 Thiverval-Grignon, France; 2 US 1426, GeT-PlaGe, Genotoul, INRA, 31326 Castanet-Tolosan, France

**Keywords:** *Brevibacterium*, Horizontal gene transfer, Comparative genomics, Cheese rind, Cheese ripening, Iron acquisition, Bacteriocin, Lanthipeptide, Lantibiotic, BreLI

## Abstract

**Background:**

*Brevibacterium* strains are widely used for the manufacturing of surface-ripened cheeses, contributing to the breakdown of lipids and proteins and producing volatile sulfur compounds and red-orange pigments. The objective of the present study was to perform comparative genomic analyses in order to better understand the mechanisms involved in their ability to grow on the cheese surface and the differences between the strains.

**Results:**

The genomes of 23 *Brevibacterium* strains, including twelve strains isolated from cheeses, were compared for their gene repertoire involved in salt tolerance, iron acquisition, bacteriocin production and the ability to use the energy compounds present in cheeses. All or almost all the genomes encode the enzymes involved in ethanol, acetate, lactate, 4-aminobutyrate and glycerol catabolism, and in the synthesis of the osmoprotectants ectoine, glycine-betaine and trehalose. Most of the genomes contain two contiguous genes encoding extracellular proteases, one of which was previously characterized for its activity on caseins. Genes encoding a secreted triacylglycerol lipase or involved in the catabolism of galactose and D-galactonate or in the synthesis of a hydroxamate-type siderophore are present in part of the genomes. Numerous Fe^3+^/siderophore ABC transport components are present, part of them resulting from horizontal gene transfers. Two cheese-associated strains have also acquired catecholate-type siderophore biosynthesis gene clusters by horizontal gene transfer. Predicted bacteriocin biosynthesis genes are present in most of the strains, and one of the corresponding gene clusters is located in a probable conjugative transposon that was only found in cheese-associated strains.

**Conclusions:**

*Brevibacterium* strains show differences in their gene repertoire potentially involved in the ability to grow on the cheese surface. Part of these differences can be explained by different phylogenetic positions or by horizontal gene transfer events. Some of the distinguishing features concern biotic interactions with other strains such as the secretion of proteases and triacylglycerol lipases, and competition for iron or bacteriocin production. In the future, it would be interesting to take the properties deduced from genomic analyses into account in order to improve the screening and selection of *Brevibacterium* strains, and their association with other ripening culture components.

**Electronic supplementary material:**

The online version of this article (10.1186/s12864-017-4322-1) contains supplementary material, which is available to authorized users.

## Background

Microbial communities from rinds of surface-ripened cheeses are composed of various bacteria, yeasts and molds, which contribute to the flavor, texture and appearance of the final products. These microorganisms may come from the milk, the ripening environment or from ripening cultures that are widely used in the cheese industry. The function of the ripening cultures is to provide specific organoleptic properties, to ensure a better regularity of manufacturing, and to outcompete pathogens or spoilage microorganisms [1]. However, strains from ripening cultures frequently do not establish themselves in cheeses [2]. Even if they are massively inoculated, these strains are sometimes outcompeted by the resident “house flora” due to insufficient fitness in the cheese surface habitat. The ability to grow on the cheese surface depends on various properties such as efficient salt tolerance and iron acquisition systems, or on the ability to use the energy compounds present in the cheese [3]. In addition, growth is influenced by the other microorganisms present at the cheese surface, with which they may have positive or negative interactions.

One example of a ripening culture component that may have problematic growth in cheeses is *Brevibacterium* [4–9]. This microorganism contributes to the breakdown of lipids and proteins, and produces volatile sulfur compounds that are key aroma impact compounds, as well as red-orange pigments [10–12]. For a long time, *Brevibacterium linens* was considered to be the major *Brevibacterium* species in cheeses. In 2004, it was broken down into three species: *B. linens*, *B. antiquum* and *B. aurantiacum* [13]. These three species, together with *B. casei* and other not-yet described *Brevibacterium* species, have been isolated from cheeses [14].

In order to improve the strategies for selecting *Brevibacterium* strains for ripening cultures, it is important to better understand the mechanisms involved in their ability to grow on the cheese surface. This can be investigated by genomic analyses. For example, the study of the genomes of the cheese strains *Glutamicibacter arilaitensis* Re117 (formerly *Arthrobacter arilaitensis* Re117) and *Corynebacterium variabile* DSM 44702 revealed several metabolic capabilities that were considered to play roles in growth on cheese [15, 16]. In addition, a recent study provided evidence of extensive horizontal gene transfer (HGT) in cheese-associated bacteria, including *Brevibacterium* strains, for which genes involved in iron acquisition were particularly abundant in the transferred islands [17].

The aim of the present study was to investigate, in *Brevibacterium* strains, key genetic determinants known to be important for growth in cheese: the catabolism of energy compounds present in cheeses, iron acquisition, salt tolerance and bacteriocin production. For that purpose, we sequenced the genome of 13 *Brevibacterium* strains, including eleven strains isolated from cheeses. We performed comparative analyses of these genomes and of ten other *Brevibacterium* genomes from strains isolated from diverse environments and already present in the Integrated Microbial Genomes (IMG) database [18].

## Methods

### Growth conditions and DNA extraction

The *Brevibacterium* strains were grown under aerobic conditions (rotary shaker at 150 rpm) for three days at 25 °C in 50-ml conical flasks containing 10 ml of brain heart infusion broth (Biokar Diagnostics, Beauvais, France). Bacterial cells were recovered by centrifugation at 4500 x g for 10 min from 5 ml of culture, washed once with 5 ml of TE buffer (Tris-HCl 10 mM, EDTA 1 mM, pH 8) and resuspended in 500 μl of the same buffer. Seventy-five μl of lysis solution containing lysozyme (40 mg/ml) and lyticase (1333 U/ml) were added and the suspensions were incubated for 30 min at 37 °C. After addition of 60 μl of 0.5 M EDTA pH 8.0, 20 μl of proteinase K (20 mg/ml) and 100 μl of 20% SDS, the samples were incubated for 1 h at 55 °C and subsequently transferred to 2-ml bead-beating tubes containing 100 mg of 0.1 mm-diameter zirconium beads and 100 mg of 0.5 mm-diameter zirconium beads. After cooling on ice, 500 μl of phenol-chloroform-isoamyl alcohol (25:24:1; saturated with 10 mM Tris, pH 8.0, and 1 mM EDTA) were added and the tubes were shaken in a bead beater (FastPrep-24, MP Biomedicals, Illkirch, France) using two 45-s mixing sequences at a speed of 6.0 m/s. The tubes were cooled on ice for 5 min after each mixing. The content of the tubes was transferred to Phase Lock Gel Heavy tubes (5 PRIME, Hilden, Germany), which were then centrifuged at 18,500 × g for 30 min at 20 °C. The aqueous phases were subsequently transferred to new Phase Lock Gel tubes. After adding 500 μl of phenol-chloroform-isoamyl alcohol and gentle mixing, centrifugation was performed at 18,500 × g for 20 min at 20 °C. Five hundred μl of chloroform were then added to the aqueous phase, and the tubes were centrifuged at 18,500 × g for 20 min at 20 °C after gently mixing. The aqueous phases (approximately 200 μl) were recovered, mixed with 1 μl of RNase A (20 mg/ml), and incubated for 30 min at 37 °C. DNA was then precipitated by adding 200 μl of cold isopropanol and the tubes were incubated for 10 min at 4 °C. The DNA was recovered by centrifugation for 15 min at 18,500 x g and 4 °C, and the pellets were subsequently washed two times with 500 μl of 70% (vol/vol) ethanol. The pellets were then dried for 15 min at 42 °C and dissolved in 100 μl of water. Finally, DNA cleanup was performed using the DNeasy Blood & Tissue Kit (Qiagen, Courtaboeuf, France) according to the manufacturer’s instructions. DNA yield and purity (absorbance ratio at 260/280 nm) were determined using a NanoDrop ND-1000 spectrophotometer (Labtech, Palaiseau, France). DNA integrity was verified by electrophoresis in a 1% agarose gel (Qbiogene, Illkirch, France) in 1X TAE buffer (40 mM Tris, 20 mM acetic acid, 1 mM EDTA, pH 8.3) stained with SYBR® Safe 1X (Invitrogen, Carlsbad, CA, USA).

### Genome sequencing, assembly and annotation

Libraries were generated using the TruSeq DNA Sample Preparation Kit (Illumina, San Diego, CA, USA) according to the manufacturer’s instructions. Sequencing was carried out on an Illumina MiSeq apparatus at the INRA GeT-PlaGE platform (http://get.genotoul.fr) in order to generate paired-end reads (250 bases in length). For each strain, the paired-end reads were merged using FLASH [19]. De novo assembly was performed using SPAdes version 3.1.1 [20]. Only contigs with length > 1000 bp were considered for further study. Gene predictions and annotations were performed automatically using the Integrated Microbial Genomes (IMG) database and comparative analysis system [21], as described in the corresponding standard operating procedure [22]. PHASTER [23] was used to predict prophages. Sequences involved in the production of secondary metabolites and bacteriocins were searched using antiSMASH 3.0 [24], BAGEL3 [25] and BACTIBASE [26].

### Comparative genomic analysis and phylogenetic classification

Comparative genomic analyses were performed considering the 13 genomes sequenced in the present study as well as the ten other *Brevibacterium* genomes present in the IMG database in September 2016 (Table 1). A phylogenetic analysis was performed for the 23 *Brevibacterium* genomes using the sequences of 40 marker genes, as described by Mende et al. [27]. The genomes of *Glutamicibacter arilaitensis* Re117 [16] (Project accession number PRJEA50353) and *Corynebacterium casei* LMG S-19264 [28] (Project accession number PRJNA186910) were used as outgroups. An in-house database of the 40 marker genes present in 388 bacterial strains, which included 117 strains isolated from dairy products [29], was used to detect the 40 marker genes in the *Brevibacterium* genomes using tBlastN [30]. The best hits were selected with at least 60% sequence identity and 80% coverage. Each marker gene was translated into an amino acid sequence using T-Coffee [31] and aligned using MUSCLE [32]. The 40 individual alignments were then concatenated to a single one, which was used to build the tree using FastTree 2 [33] with the following parameters: –gamma –pseudo –mlacc 3 –slownni and the default bootstrap procedure (1000 resamples). The tree was visualized and annotated using MEGA7 [34].

**Table 1:** Information about the *Brevibacterium* strains and genomes investigated in the present study

Species	Strain	Source	Bioproject	Sequence accession numbers	Status	Authors
*B. antiquum*	CNRZ 918	Beaufort cheese	PRJEB19830	FXZD01000001-FXZD01000049	Permanent draft	This study
*B. antiquum*	P10	Murol cheese	PRJEB19831	FXZE01000001-FXZE01000059	Permanent draft	This study
*B. aurantiacum*	ATCC 9175^T^	Camembert cheese	PRJEB19815	FXZB01000001-FXZB01000070	Permanent draft	This study
*B. aurantiacum*	CNRZ 920	Beaufort cheese	PRJEB19800	FXZG01000001-FXZG01000073	Permanent draft	This study
*B. aurantiacum*	6(3)	Langres cheese	PRJEB19867	FXYZ01000001-FXYZ01000091	Permanent draft	This study
*B. aurantiacum*	8(6)	Reblochon cheese	PRJEB19868	FXZI01000001-FXZI01000097	Permanent draft	This study
*B. casei*	CIP 102111^T^	Cheddar cheese	PRJEB19871	FXZC01000001-FXZC01000024	Permanent draft	This study
*B. linens*	ATCC 9172^T^	Harzer cheese	PRJEB19834	FXYY01000001-FXYY01000080	Permanent draft	This study
*B. linens*	Mu101	Munster cheese	PRJEB19836	FXZA01000001-FXZA01000081	Permanent draft	This study
*B. sp.*	239c	Camembert cheese	PRJEB19828	FXZH01000001-FXZH01000068	Permanent draft	This study
*B. sp.*	Mu109	Munster cheese	PRJEB19840	FXZF01000001-FXZF01000126	Permanent draft	This study
*B. iodinum*	ATCC 49514^T^	Cow milk	PRJEB19872	FXYX01000001-FXYX01000065	Permanent draft	This study
*B. jeotgali*	SJ5-8^T^	Seafood	PRJEB19841	FXZM01000001-FXZM01000047	Permanent draft	This study
*B. aurantiacum*	ATCC 9174^a^	Romadur cheese	PRJNA405	AAGP01000001-AAGP01000076	Permanent draft	DOE JGI, 2005 (direct submission)
*B. album*	DSM 18261^T^	Soil	PRJNA195785	AUFJ01000001-AUFJ01000016	Permanent draft	Kyrpides et al.*,* 2013 (direct submission)
*B. sandarakinum*	DSM 22082^T^	Wall surface	PRJEB16423	LT629739	Complete	Varghese, 2016 (direct submission) Kämpfer et al., 2010 [91]
*B. linens*	AE038–8	Fresh water	PRJNA268212	JTJZ01000001-JTJZ01000029	Permanent draft	Maizel et al.*,* 2015 [92]
*B. siliguriense*	DSM 23676^T^	Fresh water	PRJNA303729	LT629766	Complete	Varghese, 2016 (direct submission)
*B. sp.*	VCM10	Fresh water	PRJNA234061	JAJB01000001-JAJB01000141	Permanent draft	Muthukrishnan et al.*,* 2014 (direct submission)
*B. casei*	S18	Human associated	PRJNA174308	AMSP01000001-AMSP01000043	Permanent draft	Sharma et al.*,* 2012 (direct submission)
*B. ravenspurgense*	5401308^T^	Human associated	PRJNA159637	CAJD01000001-CAJD01000026	Permanent draft	Roux et al.*,* 2012 [93]
*B. mcbrellneri*	ATCC 49030^T^	Human associated	PRJNA34583	ADNU01000001-ADNU01000096	Permanent draft	Qin et al.*,* 2010 (direct submission)
*B. senegalense*	JC43^T^	Human associated	PRJEA82613	CAHK01000001-CAHK01000070	Permanent draft	Kokcha et al.*,* 2012 [94]

^a^The “*Brevibacterium linens* BL2” genome in the JGI database is in fact the genome of strain ATCC 9174 [95]

Homologous gene families were computed using the OrthoMCL procedure implemented in GET-HOMOLOGUES software [35]. Amino acid sequences of the CDSs from the 23 *Brevibacterium* genomes were grouped into clusters using 75% identity and 75% coverage thresholds with a BlastP cutoff E value <1e-05. The inflation index of OrthoMCL algorithm was set to 1.5, as recommended by Li et al. [36]. Strain ATCC 9175^T^ was set as the first reference genome, the subsequent ones were randomly chosen by GET-HOMOLOGUES. Functional category assignment to each cluster was done according to the Clusters of Orthologous Groups (COG) database [37]. The Average Nucleotide Identity (ANI), implemented in GET-HOMOLOGUES software, was computed on homologous genes for all possible pairs of genomes.

## Results

### General genomic features

Assembly and annotation metrics for the 13 newly sequenced *Brevibacterium* genomes and those concerning the ten additional genomes (which included one cheese isolate) for the comparative analysis are detailed in Additional file 1. *Brevibacterium* genomes show considerable size heterogeneity, ranging from about 2.3 to 4.5 Mbp. There is no clear relationship between genome size and the habitat from which the strains were isolated. However, genome size is quite similar among the 12 cheese isolates, 4 Mbp on average (min: 3.7; max: 4.5), and these genomes harbor an average of 3712 genes (min: 3395; max: 4154). The two smallest genomes corresponded to human-associated strains: *B. ravenspurgense* 5401308^T^ and *B. mcbrellneri* ATCC 49030^T^ (2.3 and 2.6 Mbp, respectively). The G + C content varied from 58.0% (*B. mcbrellneri* ATCC49030^T^) to 70.9% (*B. album* DSM 18261^T^), and was between 62 and 65% for most of the strains. Clustered regularly interspaced short palindromic repeat (CRISPR) candidates were found in most genomes (19 out of 23). Complete CRISPR-Cas systems consist of an array of CRISPRs interspaced by spacers and an adjacent *cas* gene cluster. Such a complete structure was observed only in the genomes of *B. casei* S18 (scaffold ID: S272_Contig15.15; 17 spacers), *B. album* DSM 18261^T^ (scaffold ID: K318DRAFT_scaffold00001.1; 2 + 1 + 6 + 35 + 12 spacers) and *B. ravenspurgense* 5401308^T^ (scaffold ID: Y1ADRAFT_CAJD01000011_1.11; 36 spacers). They all belong to the Type I CRISPR-Cas system but their overall gene content is variable. The PHASTER tool identified only one complete prophage region, in strain *B. sp.* Mu109. This 11.4 kb region (scaffold ID: Ga0063700_1029; locus tag Ga0063700_02875 to Ga0063700_02886) contains attachment sites (*attL* and *attR*) and CDSs encoding putative transposase, recombinase, integrase and capsid scaffolding proteins. However, because most genomes are draft genomes, other prophage regions might be present as regions split on several contigs and, consequently, difficult to detect.

### Phylogenomic analyses and orthology

**Fig. 1:**
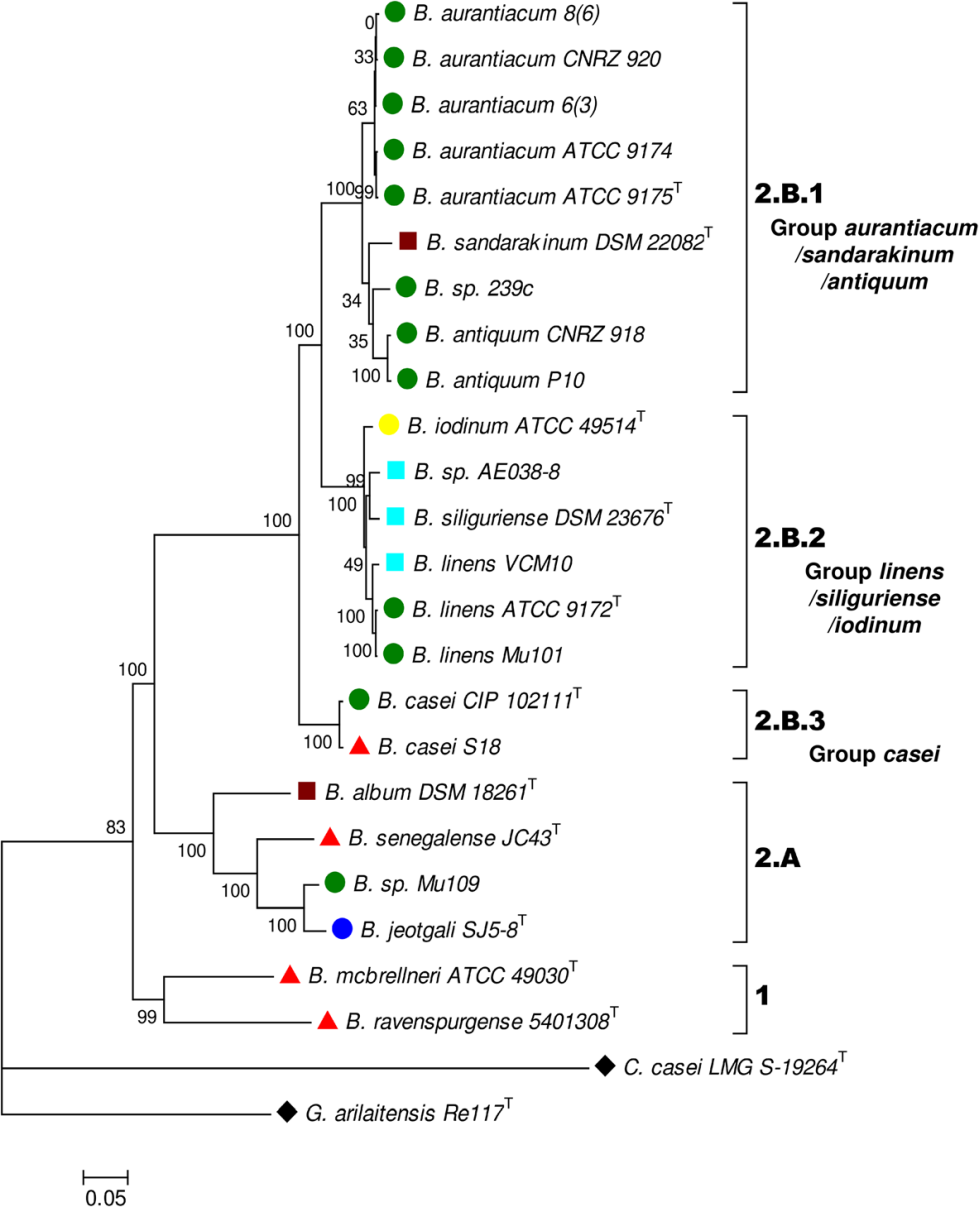
Phylogenetic tree of the 23 *Brevibacterium* genomes based on the concatenated amino acid alignments of 40 marker genes. The tree is rooted using *Glutamicibacter arilaitensis* Re117 and *Corynebacterium casei* LMG S-19264 as outgroups (). *Brevibacterium* strains are labeled according to their habitats (: cheese; : milk; : seafood; : fresh water; : soil or wall surface; : human associated). Bootstrap support values are shown as a percentage before the respective nodes; the scale bar indicates the number of substitutions per site. Phylogenetic clusters are indicated on the right of the tree

The genomic data from the 23 *Brevibacterium* strains were used to assess their intra-genus phylogenetic relationships. The phylogenomic tree partitioned the strains into two major lineages (Fig. 1). Lineage 1 contains two *Brevibacterium* strains isolated from human-associated samples, i.e., *B. mcbrellneri* ATCC49030^T^ and *B. ravenspurgense* 5401308^T^. Lineage 2, containing the 21 other strains, is composed of two branches corresponding to the groups 2.A and 2.B. Group 2.A contains four strains isolated from diverse habitats: *B. album* DSM 18261^T^ (saline soil), *B. senegalense* JC43^T^ (human stool), *B. sp.* Mu109 (cheese) and *B. jeotgali* SJ5-8^T^ (fermented seafood). Group 2.B contains all the *Brevibacterium* strains isolated from cheese except *B. sp.* Mu109. It can be divided into three clades: 2.B.1, 2.B.2 and 2.B.3. Clade 2.B.1 contains nine strains, including eight strains isolated from cheese, and *B. sandarakinum* DSM 22082^T^, which was isolated from a house wall. Clade 2.B.2 consists of six strains, including two from cheeses and three from freshwater environments. Clade 2.B.3 contains two strains belonging to the *B. casei* species, i.e., CIP 102111^T^, isolated from cheese and S18, isolated from human skin. In the following, clades 2.B.1, 2.B.2, 2.B.3 will be referred to as group “*aurantiacum/sandarakinum/antiquum*”, group “*linens/siliguriense/iodinum*” and group “*casei*”, respectively. The taxonomy of the *Brevibacterium* genus is still in process of reclassification [11]. According to Stackebrandt et al. [38], 16S rRNA (*rrs*) gene sequences and DNA-DNA hybridization (DDH) should be considered as molecular criteria for species delineation. However, the Average Nucleotide Identity (ANI) has recently been proposed to replace DDH values [39–41]. In the present study, we used two criteria for delineating species: two strains were considered to belong to the same species if (i) their *rrs* gene sequence identity was ≥98% [42] and (ii) their ANI was ≥95% [40]. The results are presented in Additional file 2. Based on these criteria, two strains isolated from cheese products, i.e. 239c and Mu109 could not be linked to any sequenced species. In addition, *B. linens* AE038–8 and *B. linens* ATCC 9172^T^ have an ANI value of only 91.2%, suggesting that strain AE038–8 does not belong to the *B. linens* species.

**Fig. 2:**
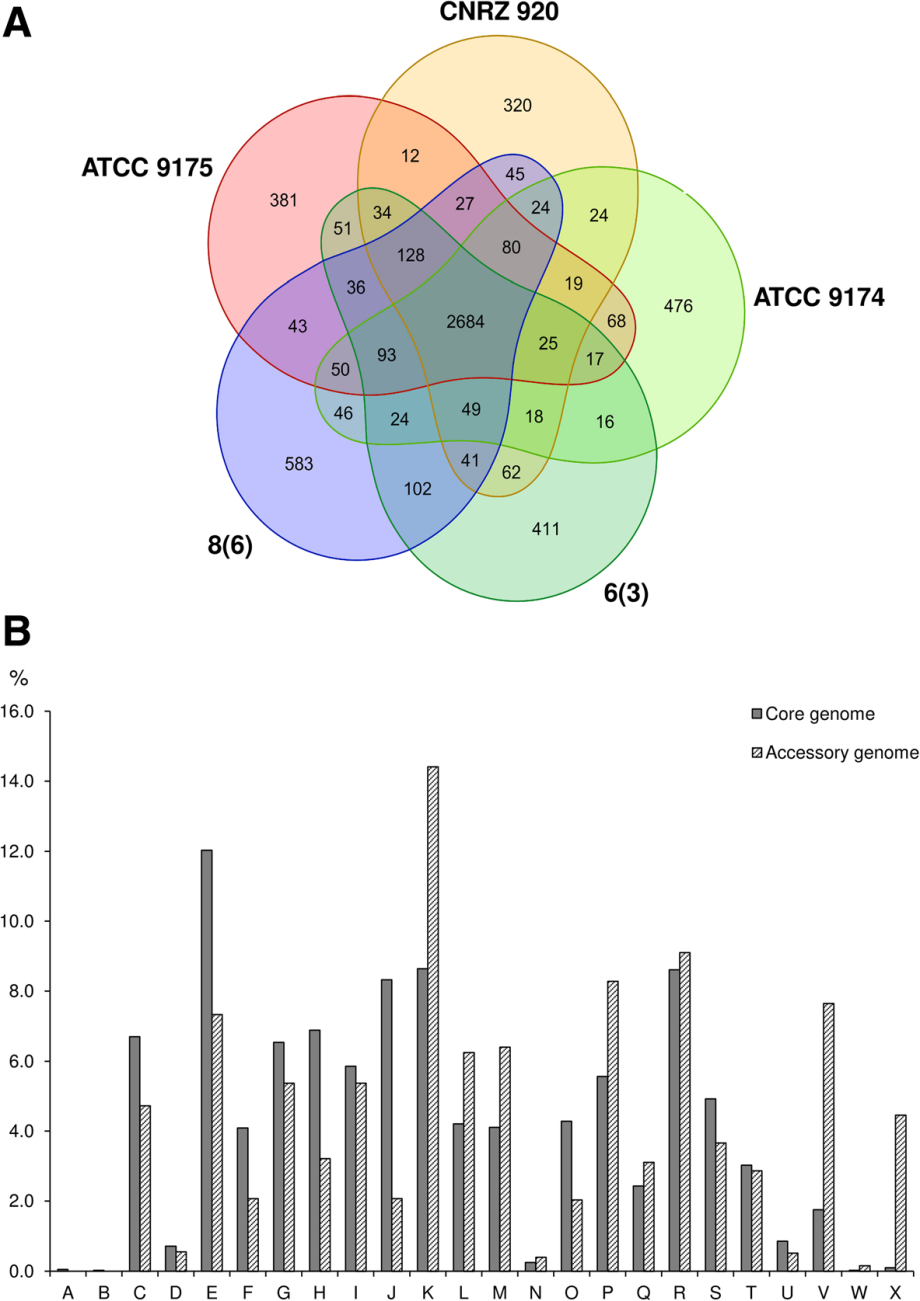
Orthologous gene clusters in the five *B. aurantiacum* genomes. **a** Venn diagram of the distribution of orthologous gene clusters. **b** Functional categories of the core and the accessory genome. Functional assignments were performed using the Integrated Microbial Genomes (IMG) platform; functional categories were labeled according to the COG database (https://www.ncbi.nlm.nih.gov/COG). A: RNA processing and modification; B: Chromatin structure and dynamics; C: Energy production and conversion; D: Cell cycle control, cell division, chromosome partitioning; E: Amino acid transport and metabolism; F: Nucleotide transport and metabolism; G: Carbohydrate transport and metabolism; H: Coenzyme transport and metabolism; I: Lipid transport and metabolism; J: Translation, ribosomal structure and biogenesis; K: Transcription; L: Replication, recombination and repair; M: Cell wall/membrane/envelope biogenesis; N: Cell motility; O: Post-translational modification, protein turnover, chaperones; P: Inorganic ion transport and metabolism; Q: Secondary metabolites biosynthesis, transport and catabolism; R: General function prediction only; S: Function unknown; T: Signal transduction mechanisms; U: Intracellular trafficking, secretion and vesicular transport; V: Defense mechanisms; X: Mobilome: prophages, transposons; W: Extracellular structures

Pan-genome analysis of the 23 *Brevibacterium* strains resulted in 25,376 orthologous gene clusters, containing a total of 78,702 protein-coding genes (Additional files 3 and 4). Of these clusters, only 263 (1%) are shared by all the strains (the core genome), reflecting a high intragenus genomic variability. We further investigated the *B. aurantiacum* species, in which all five of the sequenced strains were isolated from cheese. The pan-genome of this group contains 5988 orthologous gene clusters, of which 2684 are core and 3304 are accessory, i.e., variable among strains (Fig. 2A). Functional prediction revealed an overrepresentation of some COG categories in the accessory genome relative to the core genome, especially the categories [V] (Defense mechanisms) and [X] (Mobilome: prophages, transposons), in which the ratio accessory/core was 4.4 and 45.3, respectively (Fig. 2B).

### Catabolism of energy compounds present in cheeses

#### Catabolism of lactose, galactose and D-galactonate

During the manufacturing of cheeses, lactose is consumed by lactic acid bacteria, but some lactose may still be present at the beginning of ripening. When the lactic starter culture contains *Streptococcus thermophilus* strains, some galactose is produced from lactose, and this compound may be present in the cheese curd for several weeks [43]. No beta-galactosidase gene was identified in the 23 *Brevibacterium* genomes, which is consistent with the fact that most *Brevibacterium* strains are not able to consume lactose [11]. However, the genomes of twelve of the 23 *Brevibacterium* strains encode the four enzymes of the Leloir pathway for galactose utilization (Additional file 5). The corresponding strains belong to the phylogenetic groups *aurantiacum/sandarakinum/antiquum* and *linens/siliguriense/iodinum*. A complete pathway for D-galactonate catabolism is present in strains from the phylogenetic groups *aurantiacum/sandarakinum/antiquum*, *linens/siliguriense/iodinum* and *casei*. The genes are organized in a cluster, that encodes a transcriptional regulator, a 2-dehydro-3-deoxyphosphogalactonate aldolase [EC 4.1.2.21], a 2-dehydro-3-deoxygalactonokinase [EC 2.7.1.58], a galactonate dehydratase [EC 4.2.1.6] and one or two D-galactonate importers.

#### Catabolism of lactate, acetate, ethanol and citrate

Lactate, which is produced from lactose by lactic acid bacteria, is an important energy substrate for most aerobic cheese ripening bacteria [44]. At least two predicted lactate permease genes are present in the genomes of the 23 *Brevibacterium* strains (Additional file 5). The oxidation of lactate by lactate dehydrogenase generates pyruvate that is later catabolized through the TCA cycle. There are two types of lactate dehydrogenases in bacteria, NAD-dependant lactate dehydrogenases (nLDHs) and NAD-independent lactate dehydrogenases (iLDHs), the latter generally being considered as the enzymes responsible for lactate oxidation in bacteria [45]. Four types of iLDHs were identified in the genomes of the *Brevibacterium* strains: a quinone-dependent D-iLDH, a quinone-dependent L-iLDH, a Dld II-type quinone or cytochrome-dependent D-iLDH [46] and a three subunit quinone or cytochrome-dependent L-iLDH complex LldEFG [46]. Dld II-type quinone or cytochrome-dependent D-iLDHs were present only in group *aurantiacum/sandarakinum/antiquum*. A lactate permease gene is adjacent to the quinone-dependent L-iLDH gene in 14 strains. In cheese, acetate is produced from lactose by heterofermentative lactic acid bacteria, from citrate by citrate-utilizing lactic acid bacteria, or from lactate by *Pediococcus* and *Propionibacterium* strains. Twenty of the 23 *Brevibacterium* genomes encoded the monocarboxylic acid transporter MctC, which is an uptake system for acetate, propionate and pyruvate [47] (Additional file 5). Genes encoding acetyl-CoA synthase, which channels acetate toward the TCA cycle, are present in all the 23 strains. In cheese, ethanol is produced from lactose by yeasts such as *Kluyveromyces lactis* and *K. marxianus*, and by heterofermentative lactic acid bacteria. It is a potential energy substrate for aerobic microorganisms during cheese ripening but, to our knowledge, this has never been investigated. There are numerous candidate genes encoding enzymes involved in the catabolism of ethanol to acetate in the genomes of the *Brevibacterium* strains: between one and nine for the alcohol dehydrogenase and between nine and 31 genes for the acetaldehyde dehydrogenase (Additional file 5). In *Corynebacterium glutamicum*, the genes encoding the alcohol dehydrogenase (*adhA*) and the acetaldehyde dehydrogenase (*ald*) are responsible for ethanol catabolism [48]. All of the 23 *Brevibacterium* genomes encode an ortholog of *adhA* (65 to 69% identity at the amino acid level) and an ortholog of *ald* (67 to 82% identity), and in 21 strains, these genes form a cluster. Milk contains citrate (~8 mM in cow’s milk), which may persist during cheese ripening if the lactic starter culture does not contain citrate-utilizing lactic acid bacteria. Citrate importers belonging to the Citrate-Mg2+:H+ (CitM)/Citrate-Ca2+:H+ (CitH) Symporter (CitMHS) family (TC no. 2.A.11) and to the 2-HydroxyCarboxylate Transporter (2-HCT) family (TC no. 2.A.24) were detected in 16 *Brevibacterium* strains (Additional file 5). The genomes of the two *B. casei* strains did not encode any citrate transporter, and those of the six strains from the phylogenetic group *linens/siliguriense/iodinum* did not encode 2-HCT family citrate transporters.

#### Catabolism of lipids and glycerol

Milk contains large amounts of triglycerides (~35 g/l in cow’s milk), which can be used by cheese microorganisms as an energy source. Lipid catabolism involves the release of free fatty acids and glycerol and the subsequent breakdown of these compounds. The *Brevibacterium* genomes encode between 8 and 37 proteins with putative lipase or esterase activity (Additional file 6). Two types of secreted lipases / esterases were identified. The first type is a triacylglycerol lipase [EC 3.1.1.3], that was identified in 15 genomes, corresponding to eight of the nine strains of the phylogenetic group *aurantiacum/sandarakinum/antiquum*, to all six strains of the group *linens/siliguriense/iodinum* and to only one of the eight other strains (the cheese-associated strain Mu109). The second type is a glycerophosphoryl diester phosphodiesterase [EC 3.1.4.46], which was identified only in the strains of the phylogenetic groups *aurantiacum/sandarakinum/antiquum* and *linens/siliguriense/iodinum*. Uptake of fatty acids can be done by passive diffusion through the membrane lipid bilayer or by protein-facilitated transfer [49]. Genomic analysis revealed the presence of short-chain fatty acid uptake (AtoE) family proteins (TC no. 2.A.73) in all of the 23 *Brevibacterium* strains and it is noteworthy that *Brevibacterium* strains have a greater number of transporters from this family than other *Actinobacteria* strains (1.7 vs. 0.1 genes per genome), based on assignations to the COG2031 for the 5726 *Actinobacteria* strains present in April 2017 in the IMG database. A complete beta-oxidation pathway for fatty acid degradation was identified in all of the investigated strains, except in strains ATCC 49030^T^ and 5401308^T^, for which genes encoding L-3-hydroxyacyl-CoA dehydrogenase or 3-ketoacyl-CoA thiolase seemed to be lacking. Beta-oxidation of odd-chain-length fatty acids yields propionyl-CoA in addition to acetyl-CoA. Propionyl-CoA may be catabolized via the methylcitrate cycle, which oxidizes it to pyruvate. Three enzymes are characteristic of this cycle: the methylcitrate synthase, the methylcitrate dehydratase and the 2-methylisocitrate lyase, encoded by *prpC*, *prpD* and *prpB*, respectively [50]. In all of the investigated *Brevibacterium* genomes, these genes form a cluster (*prpDBC*). It is noteworthy that in all of the strains of the phylogenetic groups *aurantiacum/sandarakinum/antiquum*, *linens/siliguriense/iodinum* and *casei*, *prpDBC* is located upstream of the genes involved in the catabolism of glycerol (glycerol uptake protein, glycerol kinase and glycerol-3-phosphate dehydrogenase). This glycerol pathway occurs in aerobic conditions because glycerol-3-phosphate dehydrogenase reduces quinones of the respiratory chain [51]. The genome of the two *B. casei* strains also encodes a glycerol dehydrogenase and a dihydroxyacetone kinase, which constitute another glycerol utilization pathway.

#### Catabolism of proteins and amino acids

Cheese contains a large amount of proteins, mainly caseins, which can be degraded by various proteolytic microorganisms. The amino acids resulting from proteolysis can be used as an energy source by the cheese ripening microorganisms and they are also precursors of key flavor compounds. Genomic analysis of the 23 *Brevibacterium* genomes by the MEROPS peptidase BLAST search tool [52] and manual curation of the results revealed the presence of between four and 16 putative excreted enzymes with proteolytic activities, depending on the strain (Additional file 7). About 15% of these enzymes have a LPXTG motif (TIGR01167) at their C-terminus and are thus probably cell-wall-associated. Interestingly, in many cases the corresponding genes are contiguous. In addition, there was a complete identity between the sequence of one of the two predicted cell-wall-associated proteases of *B. aurantiacum* ATCC 9174 (locus tag BlinB01003410) and the first 20 N-terminal amino acid sequence of an extracellular protease purified from this strain [53]. The presence of amino acid degradation pathways was inferred from the annotation of the 23 genomes (Additional file 7). All of them encode a bifunctional proline dehydrogenase/L-glutamate gamma-semialdehyde dehydrogenase [EC 1.5.5.2 / EC 1.2.1.88], which catalyzes oxidation of proline to glutamate using a membrane-bound quinone and NAD as the electron acceptor. They also encode a NAD-specific glutamate dehydrogenase [EC 1.4.1.2] that produces NADH and alpha-ketoglutarate, an intermediate of the TCA cycle, and the enzymes involved in the degradation of threonine and serine. The histidine and alanine catabolic pathways seem to be present in all of the strains except *B. senegalense* JC43^T^ (histidine) and *B. ravenspurgense* 5401308^T^ (alanine). Phenylalanine, tyrosine, methionine and arginine degradation pathways were identified only in the strains of the phylogenetic groups *aurantiacum/sandarakinum/antiquum*, *linens/siliguriense/iodinum* and *casei*. Gamma-aminobutyrate is a four-carbon non-protein amino acid produced from glutamate by lactic acid bacteria during the ripening of cheeses [54]. All the genomes encode a 4-aminobutyrate transaminase [EC 2.6.1.19] and a succinate semialdehyde dehydrogenase [EC 1.2.1.16]. These enzymes convert Gamma-aminobutyrate into succinate, an intermediate of the TCA cycle.

### Iron acquisition

Genes encoding putative Mn^2+^ or Fe^2+^ transporters are present in all of the 23 *Brevibacterium* genomes (Additional file 8). The EfeUOB transporter, which is a high-affinity uptake system for both Fe^2+^ and Fe^3+^ [55], is present in seven strains. Five genomes encode a putative ABC-type iron transport system whose closest homolog in *Haemophilus influenza* (FbpABC) is required for acquiring iron from transferrin [56]. Fe^3+^/siderophore transport components are present in all of the 23 genomes, varying from nine components for *B. senegalense* JC43^T^, up to 31 components for *B. sp.* Mu109. Comparison of the abundance of these components based on the analysis of the COG1120, COG4604, COG0609, COG4605, COG4606, COG4779, COG0614, COG4592 and COG4607, showed that the average number was higher for *Brevibacterium* (17.6 genes per genome) than for the other *Actinobacteria* (8.4 genes per genome, calculated from 5726 *Actinobacteria* genomes). The *Brevibacterium* genomes also encode siderophore interacting proteins (mean number of 3.6 genes per genome), which are required for iron release from Fe^3+^/siderophore complexes. A putative hydroxamate-type siderophore biosynthesis cluster, which encodes a lysine N6-hydroxylase [EC 1.14.13.59], a siderophore synthetase component and, occasionally, a L-2,4-diaminobutyrate decarboxylase [EC 4.1.1.86], is present in 15 strains*.* All these strains belong to the phylogenetic groups *aurantiacum/sandarakinum/antiquum*, *linens/siliguriense/iodinum* and *casei* (Additional file 8). The genome of strain Mu109 contains a cluster (locus tag Ga0063700_02161 to Ga0063700_02193) encoding eight Fe^3+^/siderophore transport components, one siderophore interacting protein and four proteins that are probably involved in the biosynthesis of a catecholate or a mixed catecholate/hydroxamate siderophore: 4′-phosphopantetheinyl transferase EntD, MbtH protein, non-ribosomal siderophore peptide synthetase component and L-ornithine N5-oxygenase [EC 1.14.13.195]. The closest homologs of the 4′-phosphopantetheinyl transferase EntD and the non-ribosomal siderophore peptide synthetase component are found in *Streptomyces* species. The genome of strain ATCC 9174 contains a cluster (locus tag BlinB01002486 to BlinB01002496) encoding three Fe^3+^/siderophore transport components, one siderophore interacting protein, one siderophore exporter and seven genes that are probably involved in the biosynthesis of a catecholate siderophore: non-ribosomal siderophore peptide synthetase component, glycosyltransferase IroB, 2,3-dihydro-2,3-dihydroxybenzoate dehydrogenase [EC 1.3.1.28], isochorismate synthase [EC 5.4.4.2], 2,3-dihydroxybenzoate-AMP ligase [EC 2.7.7.58], isochorismatase [EC 3.3.2.1], and a putative transferase component of siderophore synthetase. These siderophore biosynthesis genes probably result from HGTs since many of their closest homologs are present, either in Gram-negative species or in *Streptomyces* or *Paenibacillus* species. In addition, comparison of the flanking regions of the siderophore biosynthesis cluster of strain ATCC 9174 to the CNRZ 920 genome (in which the cluster is absent) revealed that the cluster corresponded to an insertion that occurred in an ancestor of strain ATCC 9174 at the end of a tRNA-gly gene (locus tag BlinB_R0152 in ATCC 9174 and Ga0063691_00673 in CNRZ 920), which is followed in CNRZ 920 by a protein of unknown function (locus tag Ga0063691_00672, which is an ortholog of BlinB01002497 in ATCC 9174). For strains ATCC 9172^T^ and Mu101, there is little evidence for siderophore biosynthesis, even if it cannot be excluded, since their genome encodes a lysine decarboxylase that is clustered with a siderophore interacting protein and a Fe^3+^/siderophore binding component. With the exception of isochorismate synthase, which is an enzyme that is also involved in menaquinone biosynthesis, no genes involved in siderophore biosynthesis were identified in the genomes of strains DSM 18261^T^, SJ5-8^T^, 5401308^T^, ATCC 49030^T^ and JC43^T^. In summary, the genomic analyses indicate that 16 of the 23 *Brevibacterium* strains are probably able to produce siderophores. Hydroxamate-type siderophore genes are found in 14 strains, catecholate-type siderophore genes in strain Mu109, and both types in strain ATCC 9174. Siderophore biosynthesis is predicted to occur in all of the nine strains of the phylogenetic group *aurantiacum/sandarakinum/antiquum*, in four of the six strains of the group *linens/siliguriense/iodinum*, in the two strains of the group *casei*, but only in one of the six other *Brevibacterium* strains, which corresponded to the cheese isolate Mu109. Interestingly, analysis of the genomes present in the IMG database also revealed that four gene clusters involved in iron acquisition were shared between *Brevibacterium* strains isolated from cheeses and cheese isolates belonging to other genera (*Glutamicibacter*, *Microbacterium* and *Corynebacterium*). This corresponded to recent HGT events since the percentages of identity at the amino acid level between the genes in *Brevibacterium* and the genes in the other genera were typically ~95–100%. In most cases, transposase genes were located close to the clusters. These clusters were denoted Iron-Brev1, Iron-Brev2, Iron-Brev3 and Iron-Brev4 (Additional file 9). The cluster Iron-Brev1 corresponds to the ActinoRUSTI region that was recently described [17].

### Osmotic stress tolerance

Cheeses are salted by applying salt to their surface or by submerging them in saturated brine. One mechanism to overcome osmotic stress that results from high salt concentration is the accumulation of osmoprotectants. *Brevibacterium* strains are known to produce the osmoprotectant ectoine [57]. It is synthesized from L-aspartate-semialdehyde by the action of three enzymes: diaminobutyrate aminotransferase (EctB) [EC 2.6.1.76], diaminobutyrate acetyltransferase (EctA) [EC 2.3.1.178] and ectoine synthase (EctC) [EC 4.2.1.108]. Ectoine can be further converted to hydroxyectoine by the action of ectoine dioxygenase (EctD). Except for strains ATCC 49030^T^ and 5401308^T^, all the investigated *Brevibacterium* genomes contained the *ectABC* cluster (Additional file 10). The *ectD* gene was present in 13 of the 15 strains from the phylogenetic groups *aurantiacum/sandarakinum/antiquum* and *linens/siliguriense/iodinum*, but it was absent in the two strains of the group *casei*, and only present in one of the six other *Brevibacterium* strains. Glycine-betaine is another osmoprotectant that can be synthesized by *Brevibacterium*. The genomic analysis revealed the presence of two possible pathways. In the first one, choline is converted to glycine betaine by the combined action of a choline dehydrogenase [EC 1.1.99.1] and a betaine aldehyde dehydrogenase [EC 1.2.1.8] whereas in the second one, this conversion is catalyzed by a single enzyme, choline oxidase [EC 1.1.3.17]. Twelve of the strains have both pathways, nine have only the choline oxidase pathway, one has only the choline dehydrogenase pathway, and one has none of them. When present, the choline oxidase gene is located in a cluster containing a betaine/carnitine/choline transporter (BCCT) and a betaine-aldehyde dehydrogenase. Choline is present in milk and cheese [58], making it available for glycine-betaine biosynthesis. Interestingly the *Brevibacterium* genomes generally exhibit a greater number of BCCT than the other *Actinobacteria* genomes (6.3 vs. 1.1 genes per genome, based on assignations to COG1292) (Additional file 10). However, they do not have more ABC transport components involved in the transport of glycine betaine or related osmolytes (4.7 vs. 6.1). Trehalose is a non-reducing sugar that plays a physiological role in energy storage and also as a compatible solute [59, 60]. The genome of the 23 strains encodes a trehalose synthase [EC 5.4.99.16], which catalyzes the synthesis of trehalose from maltose (Additional file 10). Except for strain ATCC 49030^T^, trehalose can also be produced from UDP-glucose and glucose-6-phosphate via the trehalose-6-phosphate synthase [EC 2.4.1.15]/trehalose-6-phosphate phosphatase [EC 3.1.3.12] pathway. Two operons encoding multisubunit (Na^+^)(K^+^)/proton antiporters (Mrp systems) were identified in all the *Brevibacterium* genomes. These Mrp systems are composed of six subunits and the genes are organized as “group 2” *mrp* operons [61]. In other *Actinobacteria* genomes, the mean number of Mrp systems, based on assignation to COG1006, is about four times lower (0.5 vs. 2.0). The abundance of the other (Na^+^)(K^+^)/proton antiporters was similar in *Brevibacterium* and in the other *Actinobacteria* genomes (mean values of 3.9 and 4.0 genes per genome, respectively, based on assignations to the COG0025, COG1055, COG1757, COG3004, COG3067 and COG3263). It is noteworthy that *Brevibacterium* strains have a greater number of transporters from the Sodium Solute Symporter (SSS) family than other *Actinobacteria* strains (7.3 vs. 1.7 genes per genome, based on the number of proteins matching the PF00474 Hidden Markov Model).

### Bacteriocines and phenazines

**Fig. 3:**
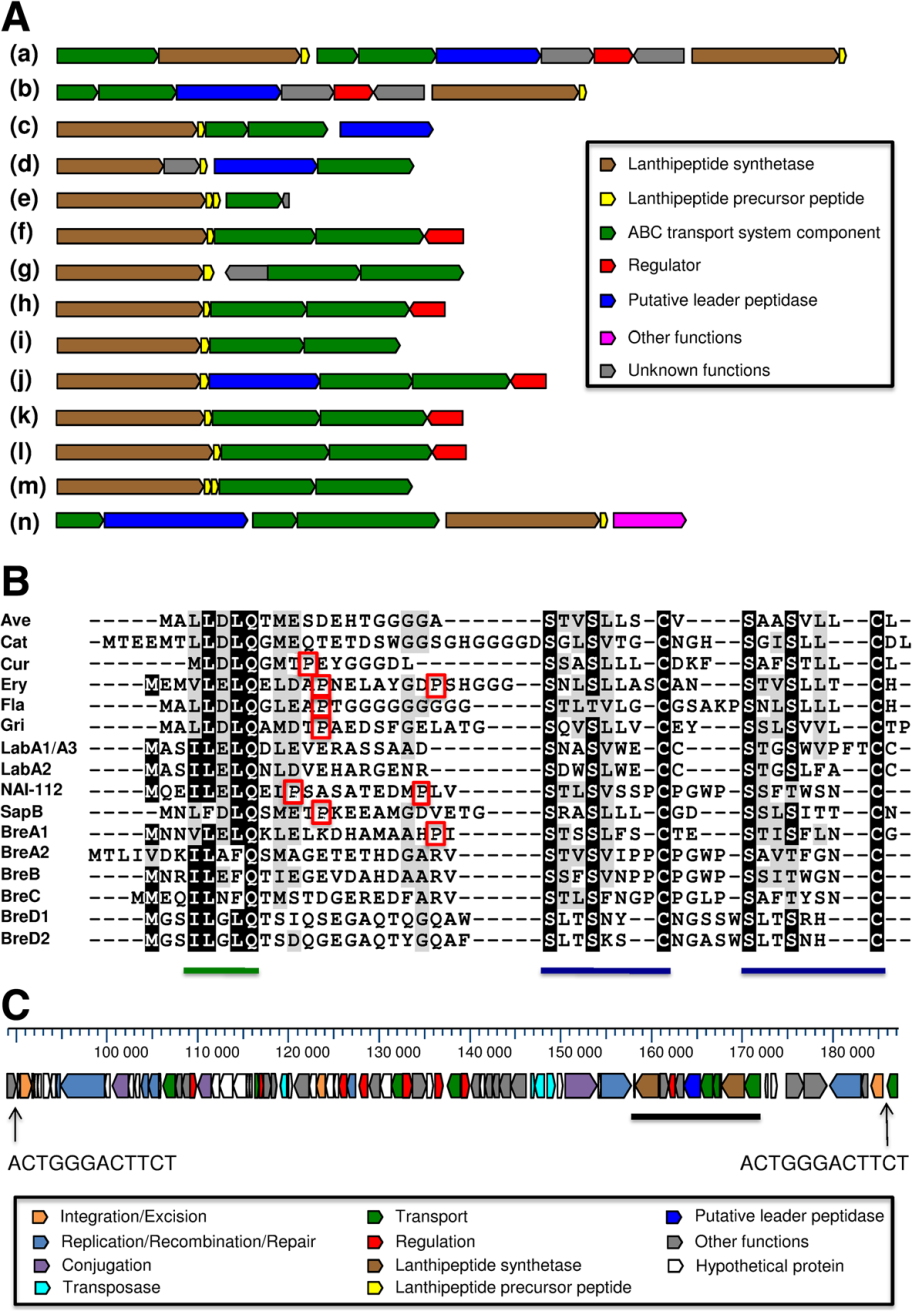
Putative lanthipeptide gene clusters in *Brevibacterium* and *Corynebacterium casei* LMG S-19264, and structure of the BreLI Island. **a** Organization of class III lanthipeptide gene clusters. (a) *B. antiquum* P10, *B. antiquum* CNRZ 918, *B. linens* ATCC 9172^T^ and *Corynebacterium casei* LMG S-19264; (b) *B. aurantiacum* ATCC 9174; (c) *B. linens* Mu101; (d) *B. mcbrellneri* ATCC 49030^T^; (e) *B. aurantiacum* CNRZ 920; (f) *Streptomyces avermitilis* MA-4680 (Avermipeptin, Ave); (g) *Catenulispora acidiphila* DSM 44928 (Catenulipeptin, Cat); (h) *Thermomonospora curvata* DSM 43183 (Curvopeptin, Cur); (i) *Saccharopolyspora erythraea* NRRL 2338 (Erythreapeptin, Ery); (j) *Kribbella flavida* DSM 17836 (Flavipeptin, Fla); (k) *Streptomyces griseus* NBRC 13350 (Griseopeptin, Gri); (l) *Streptomyces coelicolor* A3(2) (SapB); (m) *Actinomadura namibiensis* DSM 6313 (Labyrinthopeptin, Lab); (n) *Actinoplanes sp.* NAI112 (NAI-112). **b** Sequences of class III lanthipeptide precursors. (Me)Lan/(Me)Lab rings are indicated by a blue line, recognition motifs in the leader peptides are indicated by a green line, Pro residues in the leader are indicated by a red box. BreA1 corresponds to the protein product of Ga0063701_00554 (strain P10), Ga0063689_03239 (CNRZ 918), Ga0063694_02885 (ATCC 9172^T^), ORF1 (ATCC 9174, see Additional file 11 for more details), ORF25 (*Corynebacterium casei* LMG S-19264); BreA2 corresponds to the protein product of Ga0063701_00562 (P10), Ga0063689_03231 (CNRZ 918), ORF2 (ATCC 9172^T^) and ORF26 (*Corynebacterium casei* LMG S-19264); BreB, BreC, BreD1 and BreD2 correspond to the protein products of Ga0063699_00711 (Mu101), HMPREF0183_0898 (ATCC 49030^T^), Ga0063691_02687 and Ga0063691_02686 (CNRZ 920), respectively. **c** Schematic map of the BreLI Island in *B. antiquum* P10. Nucleotide position values refer to contig Ga0063701_102. The lanthipeptide gene cluster region (corresponding to BreA1 and BreA2 for strains P10, CNRZ 918, ATCC 9172^T^ and *Corynebacterium casei* LMG S-19264, and BreA1 for ATCC 9174) is underlined. The 12-bp sequences are the perfect repeat delimiting the BreLI Island

In cheese, bacteriocin producers can inhibit other microbial groups that share the same ecological niche, which confers them a selective advantage. Genomic analysis predicted the production of several bacteriocins in *Brevibacterium* strains (Additional file 11). Linocin M18-related bacteriocins (PF04454) were identified in 15 genomes, including five of the nine strains of the phylogenetic group *aurantiacum/sandarakinum/antiquum*, all six strains of the group *linens/siliguriense/iodinum* and four of the eight other strains (*B. album* DSM 18261^T^, *B. senegalense* JC43^T^, *B. jeotgali* SJ5-8^T^ and *B. sp.* Mu109). Five groups of ribosomally synthesized and post-translationally modified peptides (RiPPs) [62] were predicted in the 23 *Brevibacterium* strains. The first group corresponds to lanthipeptides, which are characterized by the presence of lanthionine (Lan) and/or methyllanthionine (MeLan) and/or labionin (Lab) residues [62, 63]. Many lanthipeptides have an antimicrobial activity, mainly against Gram-positive bacteria [64], and are referred to as lantibiotics. Lanthipeptide gene clusters were predicted in seven strains, of which six are cheese-associated (Additional file 11). Even if they have different structures, these clusters contain one or two putative lanthipeptide synthetases, one or two putative precursor peptides, one to three putative ABC transport system components and, in some cases, a prolyl oligopeptidase (PF00326) (Fig. 3A). Protein sequence analysis of the predicted lanthipeptide synthetases suggests that all of them belong to class III, corresponding to a trifunctional enzyme LanKC [65]. The sequences of all putative precursor peptides (Fig. 3B) contain two characteristic and conserved Ser/Ser(Thr)/Cys motifs necessary for the formation of (Me)Lan and/or (Me)Lab. One of them also contains a B-A-C-Leu-Gln motif in its N-terminal part (where B is Ile, Leu or Val; A is Phe or Leu; and C is Glu or Asp), which is highly conserved in class III precursor peptides and essential for the enzymatic processing of the labyrinthopeptin A2 [66]. To our knowledge, as of this time, the *Kribbella flavida* DSM 17836 protein FlaP, which is a proline-specific oligopeptidase, is the only characterized protease involved in the removal of the leader peptide of a class III lanthipeptide [67]. Genes with a weak homology with *flaP* were identified in six of the seven predicted lanthipeptide gene clusters, but only one of the six predicted precursor peptides contains a Pro residue in the leader peptide, which could serve as the primary cleavage site for the predicted peptidase (Fig. 3B). Interestingly, the putative lanthipeptide gene cluster from the four cheese-associated strains *B. antiquum* CNRZ 918, *B. antiquum* P10, *B. aurantiacum* ATCC 9174 and *B. linens* ATCC 9172^T^ is located in a ~96 kb genomic island, which is absent in the other *Brevibacterium* genomes investigated, but which is present in the genome of *Corynebacterium casei* LMG S-19264, a strain isolated from a smear-ripened cheese [28]. This genomic island, which we denoted as BreLI (**Bre**vibacterium **L**anthipeptide **I**sland), probably corresponds to an Integrative and Conjugative Element (ICE, or conjugative transposon). ICEs are typically composed of three core genetic modules involved in: (i) integration and excision; (ii) conjugation; and (iii) regulation [68]. Functional prediction of genes in BreLI revealed assignations to these three functions (Fig. 3C and Additional file 11). Integration of this ICE occurred at the 3′ end of a gene encoding a class Ib ribonucleotide reductase beta subunit, resulting in a 12-bp perfect repeat sequence, present at the two borders of BreLI. It is noteworthy that, in comparison to the four other strains, a segment containing three genes of the lanthipeptide gene cluster region is lacking in the ATCC 9174 BreLI Island (compare clusters (a) and (b) in Fig. 3A). The second group of predicted RiPPs is related to lactococcin 972 (PF09683), which has been characterized in *Lactococcus lactis* IPLA 972 [69, 70]. Genes with a weak homology with the lactococcin 972 precursor peptide were identified in six cheese-associated strains from the phylogenetic group *aurantiacum/sandarakinum/antiquum*, corresponding to two different putative prepeptides, one of them being present only in strain 239c, and the other in five *B. aurantiacum* strains (Additional file 11). In all these six strains, the bacteriocin structural gene forms an operon with three genes encoding two transmembrane proteins and a putative ABC transport systeme ATP-binding protein. Comparison of the flanking regions of the lactococcin 972-related bacteriocin biosynthesis gene cluster in strains 6(3) and 8(6) with the CNRZ 918 genome (in which the cluster is absent) revealed that the gene cluster is located in a genomic island that is inserted at the end of a tRNA-val gene (locus tag Ga0063697_02943 in strain 6(3), Ga0063698_00389 in strain 8(6) and Ga0063689_00412 in strain CNRZ 918), which is followed in CNRZ 918 by an uracil-xanthine permease (locus tag Ga0063689_00413, which is an ortholog of Ga0063697_02919 in 6(3) and Ga0063698_00365 in 8(6)). The lactococcin 972-related bacteriocin biosynthesis gene cluster in strains ATCC 9174 and CNRZ 920 is also flanked by a tRNA-val gene. The third group of predicted RiPPs corresponds to linear azol(in)e-containing peptides (LAPs), which are characterized by the presence of multiple thiazole and (methyl)oxazole heterocycles, and sometimes by their corresponding 2-electron reduced azoline state. LAP gene clusters were predicted in four *B. aurantiacum* cheese-associated strains (Additional file 11). One cluster is present in strains 6(3), ATCC 9175^T^ and ATCC 9174, and another is present only in strain 8(6). All these clusters contain putative precursor peptides, a SagB-type dehydrogenase domain-containing protein (TIGR03605), one or two YcaO cyclodehydratases (PF02624) and a cyclodehydratase-docking fusion protein (TIGR03882), representing the critical components in LAP biosynthesis [62, 71]. In addition, the gene cluster in strains ATCC 9175^T^, ATCC 9174 and 6(3) also contains a methyltransferase, which may be involved in the methylation of the peptide [72]. Comparison of the flanking regions of the predicted LAP gene cluster in strain 6(3) with the CNRZ 920 genome (in which the cluster is absent) revealed that the gene cluster is located in a genomic island whose insertion is between an aspartate racemase (orthologs with locus tag Ga0063697_02599 in strain 6(3) and Ga0063691_01440 in strain CNRZ 920) and a short-chain dehydrogenase (orthologs with locus tag Ga0063697_02614 in strain 6(3) and Ga0063691_01438 in strain CNRZ 920). The LAP gene cluster in strain 8(6) contains two putative ABC transport system components. This type of component is frequently associated with LAP biosynthesis gene clusters [62, 72]. The fourth group of predicted RiPPs corresponds to the recently discovered linaridin family, which is characterized by the presence of thioether crosslinks, like for the lanthipeptide family, but whose post-translational modifications are unrelated to the lanthipeptide biosynthesis pathway [73]. Linaridin precursor peptides were predicted in the two cheese-associated strains 239c and 8(6). These two prepeptides showed significant similarity to the characterized cypemycin (51 to 53% identity) and grisemycin (58% identity) prepeptides [73, 74]. The putative linaridin gene clusters of strains 239c and 8(6) are very similar (Additional file 11). They contain homologs of the cypemycin biosynthetic gene cluster, i.e. *cypH, cypL, cypD* and *cypT* [73]. The fifth group of predicted RiPPs is related to the sporulation delaying protein (SDP), which has been characterized in *Bacillus subtilis* [75]. SDP is produced by the *sdpABC* operon in which *sdpC* encodes the SDP precursor peptide (pro-SdpC) and *sdpA* and *sdpB* are essential for the production of the active SDP toxin [76]. Genes with a weak homology with *sdpABC* were identified in three *B. aurantiacum* cheese-associated strains (Additional file 11). Comparison of the corresponding flanking regions in strains ATCC 9175^T^ and 6(3) with the 8(6) genome (in which the cluster is absent) revealed that this gene cluster is located in a genomic island that is inserted at the end of a NAD(P)H-dependent flavin oxidoreductase (locus tag Ga0063690_01635 in strain ATCC 9175^T^, Ga0063697_00339 in strain 6(3) and Ga0063698_01092 in strain 8(6)), which is followed in 8(6) by a LuxR family transcriptional regulator (locus tag Ga0063698_01093, which is an ortholog of Ga0063690_01646 in ATCC 9175^T^ and Ga0063697_00350 in strain 6(3)).

Phenazines are heterocyclic compounds that are substituted at different points around their rings [77]. They have various colors and biological activities, such as antibiotic and intercellular signaling activities. *Brevibacterium iodinum* ATCC 49514^T^, isolated from milk, is known to produce purple extracellular crystals of the phenazine iodinin [78]. Its genome contains the *phzGFEDCB* gene cluster, which encodes one enzyme involved in chorismate synthesis (PhzG) and the five enzymes required for the generation of the “core” phenazines, phenazine-1,6- dicarboxylic acid or phenazine-1-carboxylic acid, which are precursors for strain-specific phenazine derivates (Additional file 12). This cluster is also present in four other strains, all isolated from cheeses (ATCC 9175^T^, ATCC 9172^T^, P10, and ATCC 9174), which are thus probably able to produce phenazine derivatives.

## Discussion

Microbial cultures are widely used in the cheesemaking industry. There is a high degree of expertise today in terms of the design of lactic starter cultures. This expertise includes several important properties such as the ability to grow in milk, the resistance to bacteriophages, and the generation of adequate acidifying and texturizing activities. The situation is more problematic for ripening cultures for surface-ripened cheese, which contain strains that sometimes do not grow well in cheese. This may be explained by an insufficient fitness in the cheese surface habitat in comparison to the resident “house flora”. Indeed, contrary to lactic starter cultures where growth is very fast (several hours) and facilitated by a massive inoculation in milk that contains only a low concentration of microbial cells, growth at the surface of cheeses takes a longer time (several weeks) and therefore offers more opportunities for the development of well-adapted adventitious strains.

Cheese-associated strains of *Brevibacterium* belong to different species, showing that adaptation to the cheese habitat occurred independently in different lineages. The strains sequenced in the present study belong to the species *B. aurantiacum*, *B. antiquum*, *B. linens*, *B. casei*, as well as to two not-yet described species (*B. sp*. Mu109 and 239c). In some cases, the genomes of cheese-associated strains are closely related to other strains, suggesting a recent adaptation. For example, the ANI value between strains S18 (human skin) and CIP 102111^T^ (cheese) is higher than the values between the different cheese isolates belonging to the species *B. aurantiacum* (98.22% vs. 97.48–98.04%). Interestingly, a complete prophage was found in *B. sp.* Mu109, which indicates that cheese-associated *Brevibacterium* strains may undergo phage attacks, even if, to our knowledge, there is no study in the scientific literature concerning the sensitivity to phages of ripening cultures containing *Brevibacterium* strains. It is possible that the impact of phages on these cultures has been overlooked.

In this study, genomic analyses revealed that *Brevibacterium* strains show differences in the ability to use energy compounds present in cheeses. Indeed, the galactose catabolism pathway is predicted to occur only in the phylogenetic groups *aurantiacum/sandarakinum/antiquum* and *linens/siliguriense/iodinum*, and the D-galactonate catabolism pathway only in the phylogenetic groups *aurantiacum/sandarakinum/antiquum*, *linens/siliguriense/iodinum* and *casei.* The strains from the *aurantiacum/sandarakinum/antiquum* and *linens/siliguriense/iodinum* groups have more lactate dehydrogenases than the strains from the other groups, suggesting that they have a better ability to consume lactate, which may be useful during growth in cheese. A predicted extracellular triacylglycerol lipase was found in 14 of the 15 genomes from the *aurantiacum/sandarakinum/antiquum* and *linens/siliguriense/iodinum* groups and in the cheese-associated strain Mu109, suggesting that many *Brevibacterium* strains are likely to contribute to cheese lipolysis. The resulting fatty acids constitute a potential energy source for the lipolitic *Brevibacterium* strains as well as for other microbial populations living on the cheese surface. A cluster of two genes encoding two secreted proteases with LPXTG motif is present in 16 of the 23 investigated *Brevibacterium* genomes. In *B. aurantiacum* ATCC 9174, one of these two proteases has been purified and characterized [53]. This protease is active on casein and secreted during the growth of the strain, which indicates that it probably contributes to cheese proteolysis [79, 80]. The amino acids produced from caseins and the non-protein amino acid 4-aminobutyrate are major energy substrates for the bacteria growing at the cheese surface. The 17 strains belonging to the groups *aurantiacum/sandarakinum/antiquum*, *linens/siliguriense/iodinum* and *casei* are well equipped in amino acid catabolism pathways and have a similar gene content. The six other *Brevibacterium* strains have a lower catabolic potential since they do not encode the enzymes involved in arginine, phenylalanine, methionine and tyrosine degradation.

Microorganisms growing at the cheese surface have to be able to withstand the osmotic stress due to the presence of salt. Most *Brevibacterium* genomes encode the pathways for the production of the osmoprotectants ectoine or hydroxyectoine from aspartate, glycine-betaine from choline, and trehalose. In addition, in comparison to the other *Actinobacteria* genomes, they also encode a large number of Betaine/Carnithine/Choline family Transporters (BCCT), of multisubunit (Na^+^)(K^+^)/proton antiporters, and of Sodium Solute Symporters (SSS). The two last systems take advantage of Na^+^ gradients to regulate the intracellular pH and to import nutrients. These properties probably contribute to the good resistance of *Brevibacterium* strains to the cheese salt and to their stimulation in the presence of 4% NaCl [81]. No major differences in the gene content concerning osmotic stress resistance was observed between the cheese-associated or the other strains, and between the investigated phylogenetic groups, except for *B. ravenspurgense* 5401308^T^ and *B. mcbrellneri* ATCC 49030^T^, which have a smaller number of BCCT and SSS systems. These two strains have the smallest genome sizes of the 23 investigated strains and have probably undergone a reductive genome evolution.

The present study confirms the central role of iron metabolism in bacteria from cheese surface microbial communities. *Brevibacterium* strains produce and utilize siderophores [82], and the growth of typical aerobic bacteria at the cheese surface is limited by the availability of iron [83]. The low iron content of milk, the presence of sequestering compounds such as lactoferrin, the presence of oxygen and the high pH of the cheese matrix during the growth of the acido-sensitive bacteria contribute to restricting the availability of iron. One strategy for improving iron acquisition in the cheese habitat is to produce siderophores, and this capacity can be acquired or improved by HGT. Hydroxamate-type siderophore biosynthesis seems to be widespread in *Brevibacterium* strains since gene clusters involved in their production were found in most genomes of the *aurantiacum/sandarakinum/antiquum*, *linens/siliguriense/iodinum* and *casei* groups. Clusters encoding catecholate-type siderophores were present only in two strains and resulted from horizontal transfers from other genera. Interestingly, both strains were isolated from cheese, and one of them (ATCC 9174) also possesses a gene cluster involved in the production of an acetohydroxamate-type siderophore. At least one other cheese-associated strain, *Glutamicibacter arilaitensis* Re117, has two siderophore biosynthesis clusters, including a catecholate siderophore resulting from HGT [16]. It is likely that siderophore production by cheese microorganisms also results in biotic interactions. In a recent study, it was observed that a *Staphylococcus equorum* strain, which was a weak competitor against other closely related *Staphylococcus* species in model cheese experiments, became dominant in the presence of the fungus *Scopulariopsis* [84]. This effect was attributed to fungal siderophore production, which may relieve *S. equorum* of the costly production of the siderophore staphyloferrin B and potentially provide an iron source through cross-feeding. This type of cross-feeding may also occur in microbial communities containing *Brevibacterium* strains that do not have the potential to produce siderophores, such as *B. linens* ATCC 9172^T^ and *B. linens* Mu101. The Fe^3+^/siderophore complexes are imported into the cells by ABC transport systems, whether or not these siderophores are produced by the same strain. Numerous ABC-type Fe^3+^/siderophore components are present in *Brevibacterium* strains. These systems allow the cells to take advantage of different types of siderophores available in the medium. Horizontal gene transfers concerning ABC-type Fe^3+^/siderophore components were observed in the cheese-associated strains *Glutamicibacter arilaitensis* Re117 [16], in *Corynebacterium variabile* DSM 44702 [15], and a recent study provided evidence of extensive HGTs concerning Fe^3+^/siderophore acquisition in very diverse cheese-associated bacteria [17]. One of these regions, known as ActinoRUSTI, was present in two of the 23 *Brevibacterium* strains investigated in the present study. Three other islands involved in iron acquisition that are present both in cheese-associated *Brevibacterium* strains and in cheese-associated strains belonging to other genera (*Glutamicibacter arilaitensis* Re117, *Corynebacterium casei* UCMA 3821, *Corynebacterium variabile* DSM 44702 and *Microbacterium gubbeenense* DSM 15944) were detected in the investigated genomes. Interestingly, three of these four islands were present in the cheese-associated strain *B. sp*. Mu109, which is also the strain with the highest number of ABC-type Fe^3+^/siderophore components (31 components).

Bacteriocin production is thought to play a critical role in mediating the microbial population or community interactions [85]. It may thus be assumed that it could have a significant impact on the cheese surface where microbial density may exceed 10^10^ cells per cm^2^. In the present study, we detected several putative bacteriocin gene clusters in *Brevibacterium* genomes. They corresponded to six groups of bacteriocins, i.e., linocin M18-related bacteriocins and five groups of RiPPs, and were highly variable among strains. Linocin M18, which has been characterized in the red smear cheese bacterium *B. linens* M18, is an antilisterial and wide-spectrum bacteriocin [86]. Its activity against *Listeria spp.* has also been demonstrated in a model cheese [87]. In our study, linocin M18-related bacteriocins were detected in 15 of the 23 investigated genomes, belonging to different phylogenetic groups and isolated from different habitats. This result is consistent with the fact that the structural gene *lin* encoding linocin M18 is widely distributed in coryneform bacteria [88]. To our knowledge, up until now, there has been no experimental evidence about the production of RiPPs in *Brevibacterium.* However, it cannot be excluded that some of the antibacterial substances characterized from *Brevibacterium* strains, such as the Linecin A from *B. aurantiacum* ATCC 9175^T^ [89] and/or the Linenscin OC2 from *B. linens* OC2 [90], are in fact RiPPs. Almost all the RiPP gene clusters detected in our study were found in cheese-associated strains, except for a lanthipeptide gene cluster in *B. mcbrellneri* ATCC 49030^T^, which is a human-associated strain. Interestingly, many of them seem to result from HGTs. In this study, we identified the BreLI island, which is probably a ~96-kb integrative and conjugative element (ICE) encoding for class III lanthipeptides. This island is present in four cheese-associated *Brevibacterium* strains as well as in a cheese-associated strain from another genus (*Corynebacterium casei* LMG S-19264). Taken together, our results consolidate the hypothesis that bacteriocin production may provide an ecological advantage to cheese-associated bacteria. It would be interesting in further studies to examine whether these bacteriocin gene clusters are functional, to investigate the activity spectra of these bacteriocins and to determine the influence of environmental conditions on their biosynthesis. Such information would be useful for the design of surface-ripened cheese cultures in order to improve their competitiveness against adventitious strains or to prevent growth of pathogens and spoilage microorganisms. It would also be interesting to investigate the role of phenazines, which have a broad-spectrum antibiotic activity and whose biosynthesis is predicted to occur in four cheese-associated strains. These strains belong to three different species, but the presence of the corresponding gene clusters cannot be explained by recent HGT events in view of the divergence in gene sequences.

The present study confirms that there are HGT events between microorganisms growing at the surface of cheeses. Acquisition of genes involved in siderophore biosynthesis, iron import and bacteriocin production can probably improve the fitness of the strains in the cheese habitat. It can be hypothesized that these gene transfers exert an influence on the balance between the resident “house flora” and the strains from the ripening cultures. For the latter type of strains, it is, in most cases, the same strains that are massively inoculated in all the manufacturing runs. For the adventitious “house flora”, it would be beneficial to acquire genes that improve their competitiveness to the detriment of the inoculated strains, such as genes governing bacteriocin production or iron acquisition. It would thus be interesting to examine the extent to which gene acquisition by the adventitious strains impacts the growth and stability of the components from ripening cultures used for the production of surface-ripened cheeses.

## Conclusion

Some properties deduced from genome analyses are similar in all the investigated strains, such as the ability to catabolize ethanol or 4-aminobutyrate. This is also observed for the ability to catabolize glycerol and for osmotolerant biosynthesis, except for strains 5401308^T^ and ATCC 49030^T^, which are two strains that have a smaller genome size. Other properties are mainly correlated to the phylogenetic position of the strains, whether they were isolated or not from cheese, such as the ability to catabolize galactose, lactate, amino acids, or to secrete triacylglycerol lipases. The ability to catabolize D-galactonate is present in part of the strains, and this property does not seem to be correlated to the phylogenetic position or to the habitat of the strains. There are great differences in the number of Fe^3+^/siderophore ABC transport components. Some of these genes are in clusters that are also present in cheese-associated bacteria belonging to other genera, indicating that these genes are disseminated by HGTs among strains living on the cheese surface. Two *Brevibacterium* strains isolated from cheeses also acquired a catecholate-type siderophore biosynthesis gene cluster by HGT. Bacteriocin biosynthesis genes are present in most of the strains, and one of the corresponding gene clusters is located in a probable conjugative transposon of ~96 kb (BreLI), which is present in four cheese-associated *Brevibacterium* strains as well as in *Corynebacterium casei* LMG S-19264, a strain isolated from a smear-ripened cheese. *Brevibacterium* strains thus show differences in genetic determinants involved in the growth on the cheese surface. Some of them are correlated to the phylogenetic position and others are the result of gene transfers. Part of these properties contributes to biotic interactions between strains. In the future, it would be interesting to take this information into account in order to improve the screening and selection of *Brevibacterium* strains and their association with other ripening culture components.

## Additional files

Additional file 1: Genome statistics. General features of the Brevibacterium genomes. (XLSX 15 kb)

Additional file 2: 16S–ANI. (XLSX 15 kb)

Additional file 3: Orthology. (XLSX 6663 kb)

Additional file 4: Orthology (Fig). Number of orthologous gene clusters in the 23 Brevibacterium genomes. (PDF 17 kb)

Additional file 5: Galactose-galactonate-lactate-acetate-ethanol-citrate. (XLSX 36 kb)

Additional file 6: Lipid-glycerol. (XLSX 27 kb)

Additional file 7: Excreted proteases-aminoacids. (XLSX 43 kb)

Additional file 8: Iron. (XLSX 53 kb)

Additional file 9: Iron (Fig). (PDF 98 kb)

Additional file 10: Osmotolerance. (XLSX 24 kb)

Additional file 11: Bacteriocines. (XLSX 140 kb)

Additional file 12: Phenazines. (XLSX 17 kb)

## Abbreviations

ANI: Average Nucleotide Identity
BCCT: Betaine/Carnithine/Choline family Transporter
BreLI: *Brevibacterium* Lanthipeptide Island
CDS: Coding DNA sequence
CRISPR: Clustered Regularly Interspaced Short Palindromic Repeats
DDH: DNA-DNA Hybridization
HGT: Horizontal Gene transfer
HMM: Hidden Markov Model
ICE: Integrative and Conjugative Element
iLDH: NAD-independent lactate dehydrogenase
Lab: Labionin
Lan: Lanthionine
LAP: Linear Azol(in)e-containing Peptides
MeLan: MethylLanthionine
nLDH: NAD-dependant lactate dehydrogenase
RiPP: Ribosomally synthesized and Post-translationally modified Peptide
SDP: Sporulation Delaying Protein
SSS: Sodium Solute Symporter

## References

[1] Bockelmann W, law BA, Tamime AY (2010). Secondary cheese starter cultures. Technol. Cheesemaking second Ed.

[2] Irlinger F, Layec S, Hélinck S, Dugat-Bony E (2015). Cheese rind microbial communities: diversity, composition and origin. FEMS Microbiol Lett.

[3] Monnet C, Landaud S, Bonnarme P, Swennen D (2015). Growth and adaptation of microorganisms on the cheese surface. FEMS Microbiol Lett.

[4] Brennan NM, Ward AC, Beresford TP, Fox PF, Goodfellow M, Cogan TM (2002). Biodiversity of the bacterial flora on the surface of a smear cheese. Appl Environ Microbiol.

[5] Feurer C, Vallaeys T, Corrieu G, Irlinger F (2004). Does smearing inoculum reflect the bacterial composition of the smear at the end of the ripening of a French soft, red-smear cheese?. J Dairy Sci.

[6] Goerges S, Mounier J, Rea MC, Gelsomino R, Heise V, Beduhn R (2008). Commercial ripening starter microorganisms inoculated into cheese milk do not successfully establish themselves in the resident microbial ripening consortia of a south german red smear cheese. Appl Environ Microbiol.

[7] Gori K, Ryssel M, Arneborg N, Jespersen L (2013). Isolation and identification of the microbiota of Danish farmhouse and industrially produced surface-ripened cheeses. Microb Ecol.

[8] Mounier J, Gelsomino R, Goerges S, Vancanneyt M, Vandemeulebroecke K, Hoste B (2005). Surface microflora of four smear-ripened cheeses. Appl Environ Microbiol.

[9] Rea MC, Görges S, Gelsomino R, Brennan NM, Mounier J, Vancanneyt M (2007). Stability of the biodiversity of the surface consortia of Gubbeen, a red-smear cheese. J Dairy Sci.

[10] Rattray FP, Fox PF (1999). Aspects of enzymology and biochemical properties of Brevibacterium linens relevant to cheese ripening: a review. J Dairy Sci.

[11] Forquin-Gomez M-P, Weimer BC, Sorieul L, Kalinowski J, Vallaeys T, Rosenberg E, DeLong EF, Lory S, Stackebrandt E, Thompson F (2014). The family Brevibacteriaceae. Prokaryotes Actinobacteria.

[12] Onraedt A, Soetaert W, Vandamme E (2005). Industrial importance of the genus Brevibacterium. Biotechnol Lett.

[13] Gavrish EI, Krauzova VI, Potekhina NV, Karasev SG, Plotnikova EG, Altyntseva OV (2004). Three new species of brevibacteria, Brevibacterium antiquum sp. nov., Brevibacterium aurantiacum sp. nov. and Brevibacterium permense sp. nov. Mikrobiologiia.

[14] Montel M-C, Buchin S, Mallet A, Delbes-Paus C, Vuitton DA, Desmasures N (2014). Traditional cheeses: rich and diverse microbiota with associated benefits. Int J Food Microbiol.

[15] Schröder J, Maus I, Trost E, Tauch A (2011). Complete genome sequence of Corynebacterium Variabile DSM 44702 isolated from the surface of smear-ripened cheeses and insights into cheese ripening and flavor generation. BMC Genomics.

[16] Monnet C, Loux V, Gibrat J-F, Spinnler E, Barbe V, Vacherie B (2010). The arthrobacter arilaitensis Re117 genome sequence reveals its genetic adaptation to the surface of cheese. PLoS One.

[17] Bonham KS, Wolfe BE, Dutton RJ. Extensive horizontal gene transfer in cheese-associated bacteria. elife. 2017;6 10.7554/eLife.22144 PMC5526665 28644126

[18] Markowitz VM, I-MA C, Chu K, Pati A, Ivanova NN, Kyrpides NC (2015). Ten years of maintaining and expanding a microbial genome and metagenome analysis system. Trends Microbiol.

[19] Magoč T, Salzberg SL (2011). FLASH: fast length adjustment of short reads to improve genome assemblies. Bioinforma Oxf Engl.

[20] Bankevich A, Nurk S, Antipov D, Gurevich AA, Dvorkin M, Kulikov AS (2012). SPAdes: a new genome assembly algorithm and its applications to single-cell sequencing. J. Comput. Biol. J. Comput Mol Cell Biol.

[21] Markowitz VM, I-MA C, Palaniappan K, Chu K, Szeto E, Grechkin Y (2012). IMG: the integrated microbial genomes database and comparative analysis system. Nucleic Acids Res.

[22] Huntemann M, Ivanova NN, Mavromatis K, Tripp HJ, Paez-Espino D, Palaniappan K (2015). The standard operating procedure of the DOE-JGI microbial genome annotation pipeline (MGAP v.4). Stand Genomic Sci.

[23] Arndt D, Grant JR, Marcu A, Sajed T, Pon A, Liang Y (2016). PHASTER: a better, faster version of the PHAST phage search tool. Nucleic Acids Res.

[24] Weber T, Blin K, Duddela S, Krug D, Kim HU, Bruccoleri R (2015). antiSMASH 3.0-a comprehensive resource for the genome mining of biosynthetic gene clusters. Nucleic Acids Res.

[25] van Heel AJ, de Jong A, Montalbán-López M, Kok J, Kuipers OP (2013). BAGEL3: Automated identification of genes encoding bacteriocins and (non-)bactericidal posttranslationally modified peptides. Nucleic Acids Res.

[26] Hammami R, Zouhir A, Le Lay C, Ben Hamida J, Fliss I (2010). BACTIBASE second release: a database and tool platform for bacteriocin characterization. BMC Microbiol.

[27] Mende DR, Sunagawa S, Zeller G, Bork P (2013). Accurate and universal delineation of prokaryotic species. Nat Methods.

[28] Walter F, Albersmeier A, Kalinowski J, Rückert C (2014). Complete genome sequence of Corynebacterium Casei LMG S-19264T (=DSM 44701T), isolated from a smear-ripened cheese. J Biotechnol.

[29] Almeida M, Hébert A, Abraham A-L, Rasmussen S, Monnet C, Pons N (2014). Construction of a dairy microbial genome catalog opens new perspectives for the metagenomic analysis of dairy fermented products. BMC Genomics.

[30] Altschul SF, Gish W, Miller W, Myers EW, Lipman DJ (1990). Basic local alignment search tool. J Mol Biol.

[31] Notredame C, Higgins DG, Heringa J (2000). T-coffee: a novel method for fast and accurate multiple sequence alignment. J Mol Biol.

[32] Edgar RC (2004). MUSCLE: Multiple sequence alignment with high accuracy and high throughput. Nucleic Acids Res.

[33] Price MN, Dehal PS, Arkin AP (2010). FastTree 2--approximately maximum-likelihood trees for large alignments. PLoS One.

[34] Kumar S, Stecher G, Tamura K (2016). MEGA7: Molecular Evolutionary Genetics Analysis Version 7.0 for Bigger Datasets. Mol Biol Evol.

[35] Contreras-Moreira B, Vinuesa P (2013). GET_HOMOLOGUES, a versatile software package for scalable and robust microbial pangenome analysis. Appl Environ Microbiol.

[36] Li L, Stoeckert CJ, Roos DS (2003). OrthoMCL: identification of ortholog groups for eukaryotic genomes. Genome Res.

[37] Tatusov RL, Galperin MY, Natale DA, Koonin EV (2000). The COG database: a tool for genome-scale analysis of protein functions and evolution. Nucleic Acids Res.

[38] Stackebrandt E, Frederiksen W, Garrity GM, Grimont PAD, Kämpfer P, Maiden MCJ (2002). Report of the ad hoc committee for the re-evaluation of the species definition in bacteriology. Int J Syst Evol Microbiol.

[39] Konstantinidis KT, Tiedje JM (2005). Genomic insights that advance the species definition for prokaryotes. Proc Natl Acad Sci U S A.

[40] Goris J, Konstantinidis KT, Klappenbach JA, Coenye T, Vandamme P, Tiedje JM (2007). DNA-DNA hybridization values and their relationship to whole-genome sequence similarities. Int J Syst Evol Microbiol.

[41] Richter M, Rosselló-Móra R (2009). Shifting the genomic gold standard for the prokaryotic species definition. Proc Natl Acad Sci U S A.

[42] Stackebrandt E, Goebel BM (1994). Taxonomic note: a place for DNA-DNA Reassociation and 16S rRNA sequence analysis in the present species definition in bacteriology. Int J Syst Evol Microbiol.

[43] Michel V, Martley FG (2001). Streptococcus Thermophilus in cheddar cheese--production and fate of galactose. J Dairy Res.

[44] Mounier J, Rea MC, O’Connor PM, Fitzgerald GF, Cogan TM (2007). Growth characteristics of Brevibacterium, Corynebacterium, microbacterium, and staphylococcus spp. isolated from surface-ripened cheese. Appl Environ Microbiol.

[45] Jiang T, Gao C, Ma C, Microbial XP (2014). Lactate utilization: enzymes, pathogenesis, and regulation. Trends Microbiol.

[46] Pinchuk GE, Rodionov DA, Yang C, Li X, Osterman AL, Dervyn E (2009). Genomic reconstruction of Shewanella oneidensis MR-1 metabolism reveals a previously uncharacterized machinery for lactate utilization. Proc Natl Acad Sci U S A.

[47] Jolkver E, Emer D, Ballan S, Krämer R, Eikmanns BJ, Marin K (2009). Identification and characterization of a bacterial transport system for the uptake of pyruvate, propionate, and acetate in Corynebacterium glutamicum. J Bacteriol.

[48] Auchter M, Arndt A, Eikmanns BJ (2009). Dual transcriptional control of the acetaldehyde dehydrogenase gene ald of Corynebacterium glutamicum by RamA and RamB. J Biotechnol.

[49] Hajri T, Abumrad NA (2002). Fatty acid transport across membranes: relevance to nutrition and metabolic pathology. Annu Rev Nutr.

[50] Muñoz-Elías EJ, Upton AM, Cherian J, McKinney JD (2006). Role of the methylcitrate cycle in mycobacterium tuberculosis metabolism, intracellular growth, and virulence. Mol Microbiol.

[51] Bott M, Niebisch A (2003). The respiratory chain of Corynebacterium glutamicum. J Biotechnol.

[52] Rawlings ND, Barrett AJ, Finn R (2016). Twenty years of the MEROPS database of proteolytic enzymes, their substrates and inhibitors. Nucleic Acids Res.

[53] Rattray FP, Bockelmann W, Fox PF (1995). Purification and characterization of an extracellular proteinase from Brevibacterium linens ATCC 9174. Appl Environ Microbiol.

[54] Nomura M, Kimoto H, Someya Y, Furukawa S, Suzuki I (1998). Production of gamma-aminobutyric acid by cheese starters during cheese ripening. J Dairy Sci.

[55] Miethke M, Monteferrante CG, Marahiel MA, van Dijl JM (2013). The Bacillus Subtilis EfeUOB transporter is essential for high-affinity acquisition of ferrous and ferric iron. Biochim Biophys Acta.

[56] Khan AG, Shouldice SR, Kirby SD, Yu R, Tari LW, Schryvers AB (2007). High-affinity binding by the periplasmic iron-binding protein from Haemophilus influenzae is required for acquiring iron from transferrin. Biochem J.

[57] Bernard T, Jebbar M, Rassouli Y, Himdi-Kabbab S, Hamelin J, Blanco C (1993). Ectoine accumulation and osmotic regulation in Brevibacterium linens. Microbiology.

[58] Zeisel SH, Mar M-H, Howe JC, Holden JM (2003). Concentrations of choline-containing compounds and betaine in common foods. J Nutr.

[59] Frings E, Kunte HJ, Galinski EA (1993). Compatible solutes in representatives of the genera Brevibacterium and Corynebacterium: occurrence of tetrahydropyrimidines and glutamine. FEMS Microbiol Lett.

[60] Kempf B, Bremer E (1998). Uptake and synthesis of compatible solutes as microbial stress responses to high-osmolality environments. Arch Microbiol.

[61] Swartz TH, Ikewada S, Ishikawa O, Ito M, Krulwich TA (2005). The Mrp system: a giant among monovalent cation/proton antiporters. Extremophiles.

[62] Arnison PG, Bibb MJ, Bierbaum G, Bowers AA, Bugni TS, Bulaj G (2013). Ribosomally synthesized and post-translationally modified peptide natural products: overview and recommendations for a universal nomenclature. Nat Prod Rep.

[63] Meindl K, Schmiederer T, Schneider K, Reicke A, Butz D, Keller S (2010). Labyrinthopeptins: a new class of carbacyclic lantibiotics. Angew Chem Int Ed Engl.

[64] Bierbaum G, Sahl H-G (2009). Lantibiotics: mode of action, biosynthesis and bioengineering. Curr Pharm Biotechnol.

[65] Zhang Q, Yu Y, Vélasquez JE, van der Donk WA (2012). Evolution of lanthipeptide synthetases. Proc Natl Acad Sci U S A.

[66] Müller WM, Ensle P, Krawczyk B, Süssmuth RD (2011). Leader peptide-directed processing of labyrinthopeptin A2 precursor peptide by the modifying enzyme LabKC. Biochemistry (Mosc).

[67] Völler GH, Krawczyk B, Ensle P, Süssmuth RD (2013). Involvement and unusual substrate specificity of a prolyl oligopeptidase in class III lanthipeptide maturation. J Am Chem Soc.

[68] Bi D, Xu Z, Harrison EM, Tai C, Wei Y, He X (2012). ICEberg: a web-based resource for integrative and conjugative elements found in bacteria. Nucleic Acids Res.

[69] Martínez B, Fernández M, Suárez JE, Rodríguez A (1999). Synthesis of lactococcin 972, a bacteriocin produced by Lactococcus lactis IPLA 972, depends on the expression of a plasmid-encoded bicistronic operon. Microbiol Read Engl.

[70] Sánchez C, Hernández de Rojas A, Martínez B, Argüelles ME, Suárez JE, Rodríguez A (2000). Nucleotide sequence and analysis of pBL1, a bacteriocin-producing plasmid from Lactococcus lactis IPLA 972. Plasmid.

[71] Haft DH, Basu MK, Mitchell DA (2010). Expansion of ribosomally produced natural products: a nitrile hydratase- and Nif11-related precursor family. BMC Biol.

[72] Lee SW, Mitchell DA, Markley AL, Hensler ME, Gonzalez D, Wohlrab A (2008). Discovery of a widely distributed toxin biosynthetic gene cluster. Proc Natl Acad Sci U S A.

[73] Claesen J, Bibb M (2010). Genome mining and genetic analysis of cypemycin biosynthesis reveal an unusual class of posttranslationally modified peptides. Proc Natl Acad Sci U S A.

[74] Claesen J, Bibb MJ (2011). Biosynthesis and regulation of grisemycin, a new member of the linaridin family of ribosomally synthesized peptides produced by Streptomyces Griseus IFO 13350. J Bacteriol.

[75] Liu W-T, Yang Y-L, Xu Y, Lamsa A, Haste NM, Yang JY (2010). Imaging mass spectrometry of intraspecies metabolic exchange revealed the cannibalistic factors of Bacillus Subtilis. Proc Natl Acad Sci U S A.

[76] Pérez Morales TG, Ho TD, Liu W-T, Dorrestein PC, Ellermeier CD (2013). Production of the cannibalism toxin SDP is a multistep process that requires SdpA and SdpB. J Bacteriol.

[77] Price-Whelan A, Dietrich LEP, Newman DK (2006). Rethinking “secondary” metabolism: physiological roles for phenazine antibiotics. Nat Chem Biol.

[78] Whitman W, Goodfellow M, Kämpfer P, Busse H-J, Trujillo M, Ludwig W (2012). Bergey’s manual of systematic bacteriology (second edition) volume 5: the Actinobacteria.

[79] Rattray FP, Fox PF, Healy A (1996). Specificity of an extracellular proteinase from Brevibacterium linens ATCC 9174 on bovine alpha s1-casein. Appl Environ Microbiol.

[80] Rattray FP, Fox PF, Healy A (1997). Specificity of an extracellular proteinase from Brevibacterium linens ATCC 9174 on bovine beta-casein. Appl Environ Microbiol.

[81] Masoud W, Jakobsen M (2005). The combined effects of pH, NaCl and temperature on growth of cheese ripening cultures of Debaryomyces Hansenii and coryneform bacteria. Int Dairy J.

[82] Noordman WH, Reissbrodt R, Bongers RS, Rademaker JLW, Bockelmann W, Smit G (2006). Growth stimulation of Brevibacterium sp. by siderophores. J Appl Microbiol.

[83] Monnet C, Back A, Irlinger F (2012). Growth of aerobic ripening bacteria at the cheese surface is limited by the availability of iron. Appl Environ Microbiol.

[84] Kastman EK, Kamelamela N, Norville JW, Cosetta CM, Dutton RJ, Wolfe BE (2016). Biotic interactions shape the ecological distributions of staphylococcus species. MBio.

[85] Riley MA, Wertz JE (2002). Bacteriocins: evolution, ecology, and application. Annu Rev Microbiol.

[86] Valdés-Stauber N, Scherer S (1994). Isolation and characterization of Linocin M18, a bacteriocin produced by Brevibacterium linens. Appl Environ Microbiol.

[87] Eppert I, Valdés-Stauber N, Götz H, Busse M, Scherer S (1997). Growth reduction of listeria spp. caused by undefined industrial red smear cheese cultures and bacteriocin-producing Brevibacterium linens as evaluated in situ on soft cheese. Appl Environ Microbiol.

[88] Valdes-Stauber N, Scherer S (1996). Nucleotide sequence and taxonomical distribution of the bacteriocin gene lin cloned from Brevibacterium linens M18. Appl Environ Microbiol.

[89] Kato F, Eguchi Y, Nakano M, Oshima T, Murata A (1991). Purification and characterization of Linecin-a, a Bacteriocin of *Brevibacterium linens*. Agric Biol Chem.

[90] Maisnier-Patin S, Richard J (1995). Activity and purification of linenscin OC2, an antibacterial substance produced by Brevibacterium linens OC2, an orange cheese coryneform bacterium. Appl Environ Microbiol.

[91] Kämpfer P, Schäfer J, Lodders N, Busse H-J (2010). Brevibacterium sandarakinum sp. nov., isolated from a wall of an indoor environment. Int J Syst Evol Microbiol.

[92] Maizel D, Utturkar SM, Brown SD, Ferrero MA, Rosen BP (2015). Draft genome sequence of Brevibacterium linens AE038-8, an extremely arsenic-resistant bacterium. Genome Announc.

[93] Roux V, Robert C, Gimenez G, Raoult D (2012). Draft genome sequence of Brevibacterium massiliense strain 541308T. J Bacteriol.

[94] Kokcha S, Ramasamy D, Lagier J-C, Robert C, Raoult D, Fournier P-E (2012). Non-contiguous finished genome sequence and description of Brevibacterium senegalense sp. nov. Stand Genomic Sci.

[95] Ganesan B, Seefeldt K, Weimer BC (2004). Fatty acid production from amino acids and alpha-keto acids by Brevibacterium linens BL2. Appl Environ Microbiol.

