# Supplementary figures and images for: Comparative genomic analysis of Brevibacterium strains: insights into key genetic determinants involved in adaptation to the cheese habitat
Source: BMC Genomics. 2017 Dec 7;18:955. doi: 10.1186/s12864-017-4322-1 (PMC5719810; doi:10.1186/s12864-017-4322-1)

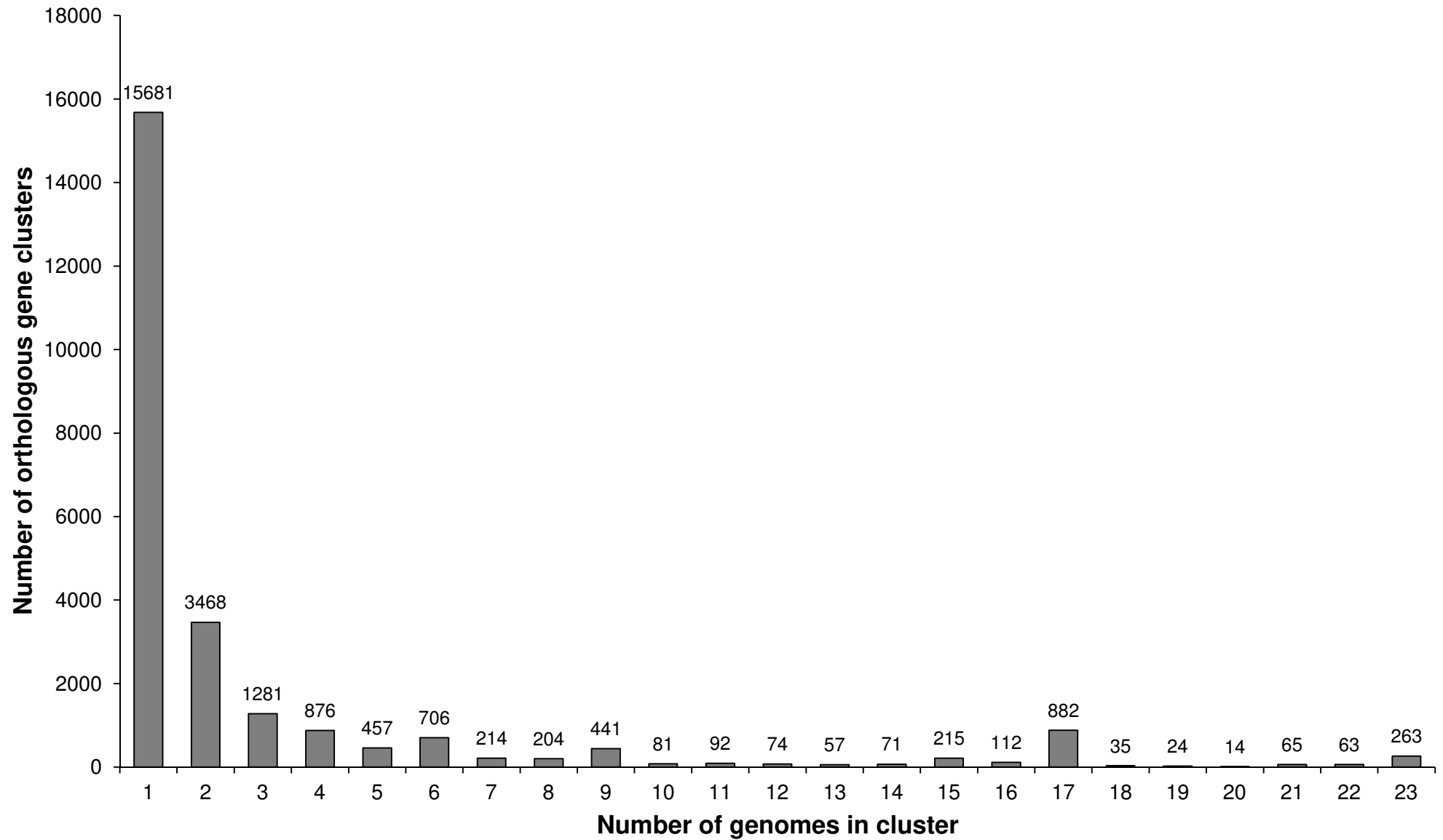

Number of orthologous gene clusters in the 23 *Brevibacterium* genomes

Supplement: Supplementary file 4 — Orthology (Fig). Number of orthologous gene clusters in the 23 Brevibacterium genomes. (PDF 17 kb) [file 12864_2017_4322_MOESM4_ESM.pdf]
