# Supplementary material for: Comparative genomic analysis of Brevibacterium strains: insights into key genetic determinants involved in adaptation to the cheese habitat
Source: BMC Genomics. 2017 Dec 7;18:955. doi: 10.1186/s12864-017-4322-1 (PMC5719810; doi:10.1186/s12864-017-4322-1)
Supplement: Supplementary file 9 — Iron (Fig). (PDF 98 kb) [file 12864_2017_4322_MOESM9_ESM.pdf]

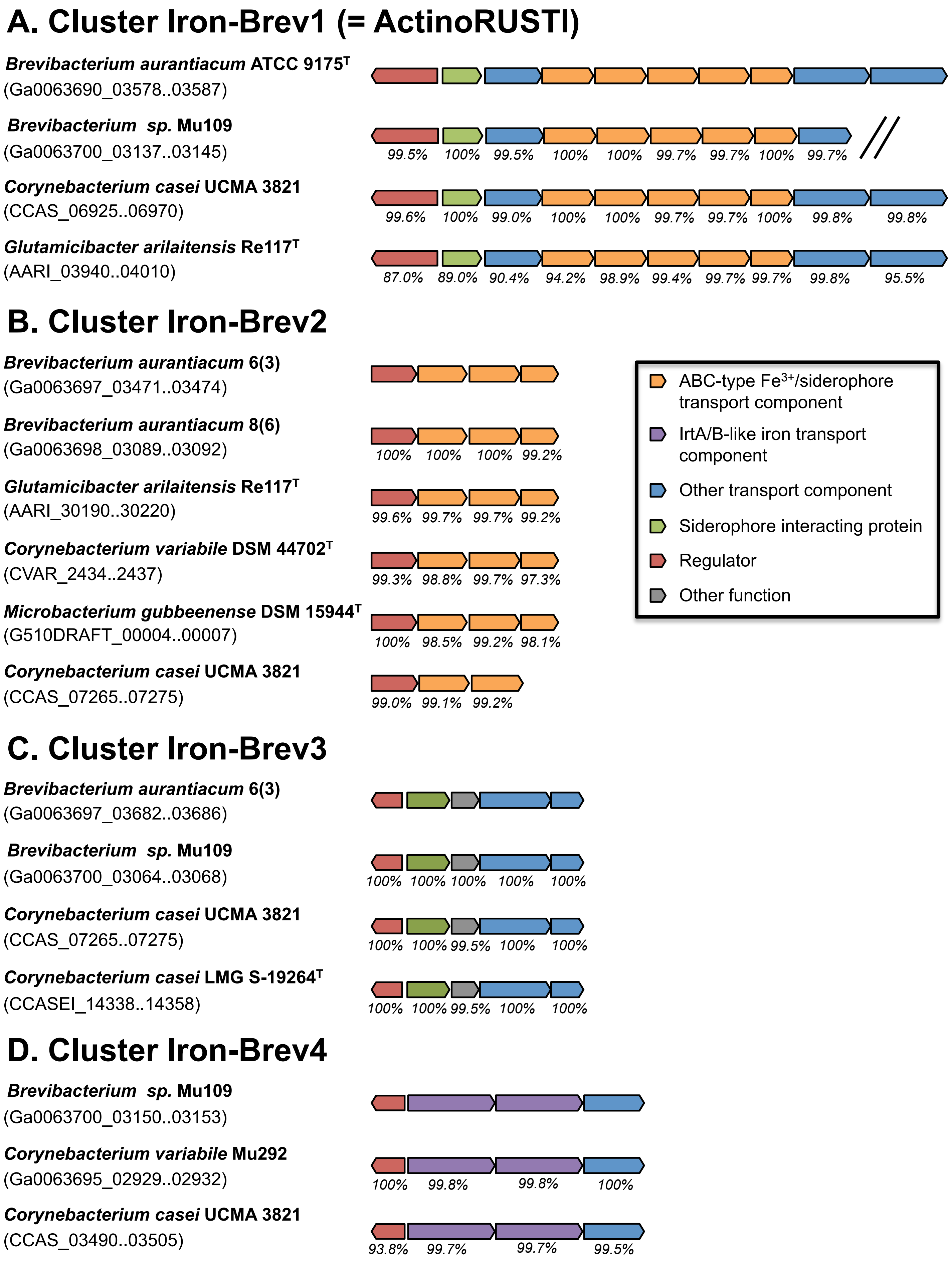

Gene clusters shared between *Brevibacterium* strains isolated from cheeses and cheese-associated strains belonging to other genera. Gene locus tags are indicated in parentheses, percentages of amino acid identity are indicated in italics.
